# Oxytocin Improves Autistic Behaviors by Positively Shifting GABA Reversal Potential via NKCC1 in Early‐Postnatal‐Stage

**DOI:** 10.1002/advs.202415432

**Published:** 2025-04-30

**Authors:** Zi‐Hui Wang, Chang Xu, Yao‐Yao Ma, Wei‐Xuan Xue, Hao‐Yuan Wang, Lin‐Yao Fan, Chen‐Yu Zhang, Liang Li, Xiao‐Yang Zhang, Jing‐Ning Zhu, Qi‐Peng Zhang

**Affiliations:** ^1^ Nanjing Drum Tower Hospital Center of Molecular Diagnostic and Therapy State Key Laboratory of Pharmaceutical Biotechnology School of Life Sciences Nanjing University Nanjing Jiangsu 210023 China; ^2^ Institute for Brain Sciences Nanjing University Nanjing Jiangsu 210023 China; ^3^ Jiangsu Engineering Research Center for MicroRNA Biology and Biotechnology NJU Advanced Institute of Life Sciences (NAILS) Nanjing Jiangsu 210023 China; ^4^ NJU Institute of AI Biomedicine and Biotechnology Nanjing University Nanjing Jiangsu 210023 China; ^5^ Research Unit of Extracellular RNA Chinese Academy of Medical Sciences Nanjing Jiangsu 210023 China; ^6^ Chemistry and Biomedicine Innovation Center (ChemBIC) ChemBioMed Interdisciplinary Research Center Nanjing University Nanjing Jiangsu 210023 China; ^7^ Department of Gastroenterology Nanjing Drum Tower Hospital Affiliated Hospital of Medical School Nanjing University Nanjing Jiangsu 210008 China

**Keywords:** autism, early postnatal stage, GABA reverse potential, NKCC1, oxytocin

## Abstract

Accumulating evidence has identified disrupted oxytocin signaling in both autistic patients and animal models of autism. Nevertheless, the specific timing of the impact of oxytocin on social behavior has remained unclear. Using mouse strains from oxytocin‐Cre mice crossed with Cre‐dependent chemogenetic mice, oxytocinergic neuronal activity is selectivity manipulated during the early or late postnatal stages and revealed, for the first time, that the suppression of oxytocinergic neurons in the early rather than late postnatal stage led to the emergence of autistic‐like behaviors. Notably, significantly reduced oxytocin levels are identified specifically during the early postnatal stage in both valproic acid (VPA)‐exposed and *Fmr1*‐KO mouse brains, along with an impairment of the GABA reversal potential and downregulation of the Na^+^‐K^+^‐2Cl^−^ cotransporter (NKCC1) post‐birth. Furthermore, chemogenetic activation of oxytocinergic neurons during the early rather than late postnatal stage effectively restored the aberrant NKCC1 expression and GABA_A_ receptor reversal potential and consequently alleviated autistic‐like behaviors in VPA‐exposed mice. Overall, the results demonstrate that the early postnatal stage may be the unique critical period for oxytocin signaling to regulate GABA reversal potential and promote brain development for prosocial behaviors. These findings suggest an earlier intervention window and strategy for the clinical oxytocin treatment of autism.

## Graphical abstract

New research reveals that oxytocin signaling during the early postnatal period is critical for regulating brain development and social behavior. Selectively suppressing oxytocin neurons by chemogenetic method shows that early—but not late—disruption leads to autism‐like behaviors. Restoring oxytocin activity during this window corrected brain imbalances and improved social behavior, suggesting an optimal intervention period for autism treatment.

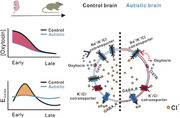

## 1 Introduction

Both genetic and environmental factors play important roles in the development of autism spectrum disorders (ASDs),^[^ ^1^ ^]^ particularly during the critical period of brain development around birth, and include prenatal infections, exposure to valproic acid (VPA), and birth complications.^[^ ^2^ ^]^ Among the various potential therapies, a particularly intriguing line of research has focused on the role of oxytocin because of its involvement in social behavior development and social recognition memory.^[^ ^3^ ^]^ Following birth, a diverse array of sensory stimuli is instrumental in shaping the establishment of functional neural circuits that underlie social behaviors, potentially mediated by oxytocin in an experience‐dependent, cross‐modal manner.^[^ ^4^ ^]^ The foundational establishment of the mother‐infant bond during this early developmental phase strongly affects the subsequent social interactions of offspring.^[^ ^5^ ^]^

Both oxytocin‐knockout (KO) and oxytocin receptor (OXTR)‐KO mice displayed deficits in social interaction.^[^ ^6^ ^]^ Importantly, studies conducted with animal models of autism have further explored the relationship between oxytocin and social behavior. For example, Peñagarikano et al. reported decreased oxytocin levels in *Cntnap2*‐mutated mice, with oxytocin administration rescuing altered social behavior in this autistic model.^[^ ^7^ ^]^ Similarly, neonatal *Magel2*‐KO mice exhibited oxytocin deficiency, and early oxytocin treatment ameliorated these social deficits.^[^ ^8^ ^]^ Moreover, sensory‐deprived mice, such as those subjected to early‐life bilateral whisker trimming or dark rearing, presented reduced brain oxytocin levels.^[^ ^9^ ^]^ In POGZ^WT/Q1038R^ mice, low expression of the OXTR gene was observed, with intranasal oxytocin administration effectively normalizing impaired social behavior.^[^ ^10^ ^]^ These findings indicate oxytocin system impairment during postnatal brain development in autistic individuals and emphasize the substantial impact of oxytocin signal dysfunction on social performance.^[^ ^11^ ^]^ ASD is a pervasive neurodevelopmental disorder that manifests symptoms in early infancy, with the typical onset occurring by the age of three. Therefore, investigations into possible treatments for autism should encompass the neurodevelopmental continuum, incorporating both parturition and early postnatal development. Importantly, a random clinical trial reported that early intervention with oxytocin can effectively increase caregiver‐rated social responsiveness (SRS‐2).^[^ ^12^ ^]^ These findings indicate that oxytocin influences brain development and social behavior in the early postnatal development stage.

During early postnatal development, a biologically conserved phenomenon known as the GABA polarity switch plays a pivotal role in nervous system development, encompassing processes such as proliferation, migration, and differentiation.^[^ ^13^ ^]^ Notably, a transient GABA polarity switch occurs rapidly during delivery, followed by a rebound to the excitatory state after birth, conferring crucial protective effects during the stressful experience of childbirth.^[^ ^14^ ^]^ Dysfunction in this GABA polarity switch has been observed in autistic brains, with maternal oxytocin regulating the switch by modulating the Na^+^‐K^+^‐2Cl^−^ cotransporter (NKCC1), which controls chloride homeostasis and the GABA reversal potential.^[^ ^15^ ^]^ Intriguingly, the above study also revealed notable abnormalities in GABA_A_ receptor reversal potential (more negative) in the brains of VPA‐exposed rats at the early postnatal stage (P2). These rapid and dynamic changes in GABA_A_ receptor reversal potential during early postnatal development suggest the potential importance of this period for the initiation of the effects of oxytocin on the GABA polarity switch in pups. However, after birth, there was no additional supply of maternal oxytocin, and whether the pup's endogenous oxytocin modulates the GABA polarity switch remains unexplored.

To investigate the critical period during which oxytocin shapes social behavior and particularly modulates GABA_A_ receptor reversal potential, we manipulated oxytocin levels during different developmental stages by using a chemogenetic technique known as designer receptors exclusively activated by designer drugs (DREADDs). We focused on the first week after birth (P1–P7) as the early postnatal developmental stage and the third week after birth (P21–P27) as the late postnatal developmental stage. By comparing the effects of manipulating oxytocinergic neuron activities at these two stages, we found that the inhibition of oxytocinergic neurons during the early rather than the late postnatal stage resulted in autistic‐like behaviors in adolescent mice. In contrast, the activation of oxytocinergic neurons during the same period mitigated abnormalities in VPA‐exposed autistic mice. Furthermore, VPA‐exposed brains presented lower levels of NKCC1 and reduced GABA reversal potential, which could be rescued by activating oxytocinergic neurons during the early but not late postnatal stage. These results demonstrate that early postnatal development is crucial for endogenous oxytocin to regulate GABA reversal potential and shape social behavior.

## 2 Results

### 2.1 Chemogenetic Inhibition of Oxytocinergic Neurons During Early Postnatal Stage Resulted in Autism‐Like Behaviors

We recently generated a novel strain of oxytocin‐knockout (Oxt‐KO) mice using CRISPR/Cas9 technology, in which a 6850 bp segment encompassing all three exons of the oxytocin gene was deleted.^[^ ^16^ ^]^ Previous studies have demonstrated that knocking out the first exon of the oxytocin gene in mice leads to social behavior abnormalities. Specifically, infant Oxt‐KO mice exhibit reduced vocalizations compared to wild‐type (WT) mice, and obligate oxytocin KO mice display aggressive behaviors.^[^ ^6^, ^17^ ^]^ To determine whether this novel Oxt‐KO strain also exhibits social deficits, we systematically characterized the social, repetitive, and general locomotor behaviors of this new strain of oxytocin‐KO mouse and observed typical autistic‐like behaviors in these oxytocin‐KO mice, which confirmed the essential role of oxytocin in social behavior development (Figure S1, Supporting Information).

**Figure 1:**
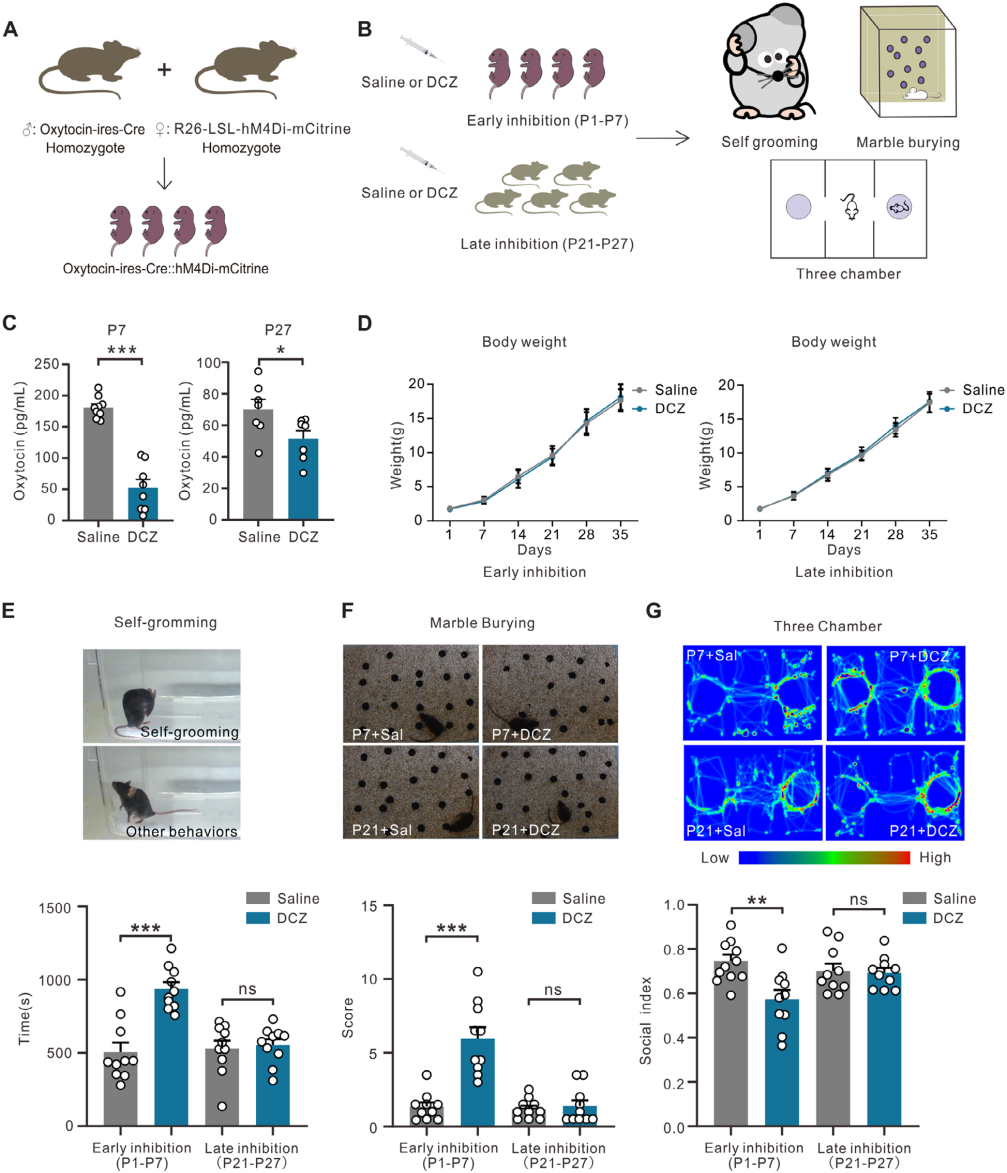
Early postnatal inhibition of oxytocinergic neurons caused autism‐like behaviors. (A) Oxytocin‐IRES‐Cre male mice were crossed with Rosa26 LSL‐hM4Di‐mCistine female mice to obtain Oxytocin‐IRES‐Cre::Rosa26 LSL‐hM4Di‐mCistine (Oxt‐hM4Di) pups. (B) The paradigm of OXT system inhibition at different postnatal stages. Three behavioral tests were carried out at intervals of 5 days from P30. (C) Hippocampal oxytocin contents after deschloroclozapine (DCZ) injection, at P7 (saline *n* = 9, DCZ *n* = 8, unpaired *t*‐test with Welch's correction, ***p < 0.001), and at P27 (*n* = 7, unpaired *t*‐test with Welch's correction, *p < 0.05). (D) Growth of the pup body weight [early inhibition (*n* = 10), P1 *p* > 0.9999, P7 *p* = 0.9998, P14 *p* = 0.9845, P21 *p* = 0.9989, P28 *p* = 0.9957, P35 *p* = 0.9701; late inhibition (*n* = 10), P1 *p* > 0.9999, P7 *p* > 0.9999, P14 *p* = 0.9941, P21 *p* = 0.9854, P28 *p* = 0.6290, P35 *p* = 0.9997]. (E) Time spent self‐grooming [early inhibition (*n* = 10), unpaired *t*‐test, ***p < 0.001; late inhibition (*n* = 10), unpaired *t*‐test, ns, *p* = 0.7243]. (F) In the marble‐burying test, mice obtained higher scores after burying more marbles [early inhibition (*n* = 10), unpaired *t*‐test, ***p < 0.001; late inhibition (*n* = 10), unpaired *t*‐test, ns, *p* = 0.6556]. (G) Three‐chamber social preference test [early inhibition (*n* = 10), unpaired *t*‐test, **p < 0.01; late inhibition (*n* = 10), ns, unpaired *t*‐test, *p* = 0.8201].

To precisely delineate the time window in which oxytocin exerts its prosocial effects, we crossbred Oxytocin‐IRES‐Cre mice with Rosa26 LSL‐hM4Di‐mCitrine mice to generate Oxt‐IRES‐Cre::hM4Di‐mCitrine mice (**Figure** **1**A). In these Oxt‐IRES‐Cre::hM4Di‐mCitrine (Oxt‐hM4Di) mice, the green fluorescence signal of hM4Di‐mCitrine was exclusively localized to oxytocin‐positive neurons (Figure S2, Supporting Information), thereby confirming the neuronal specificity of hM4Di‐mCitrine expression. The mice with the correct genotype were subjected to saline or deschloroclozapine (DCZ) treatment during early (P1, P3, P5, and P7) or late (P21, P23, P25, and P27) developmental stages to specifically inhibit oxytocinergic neurons, and their behaviors were assessed at P30 (Figure 1B). Enzyme‐linked immunosorbent assay (ELISA) results confirmed a significant reduction in oxytocin in the Oxt‐hM4Di pup brains at both P7 and P27 following DCZ treatment (Figure 1C). The body weights of the pups were measured, as shown in Figure 1D, and no significant alterations were observed upon the inhibition of oxytocinergic neurons during the early or late stages.

We next analyzed their behavioral phenotypes. The results revealed that the mice with early postnatal oxytocinergic neuron inhibition exhibited increased repetitive behaviors, evidenced by self‐grooming time rising from 505.5 ± 64.5 s in the saline group to 937.0 ± 46.3 s in the DCZ‐treated group (*p* < 0.001, Figure 1E). Additionally, marble‐burying activities were also significantly increased in the early inhibition group: from 1.35 ± 0.29 marbles in the saline group to 5.95 ± 0.78 marbles in the DCZ‐treated group (*p* < 0.001, Figure 1F). Social interaction deficits were observed in the same early inhibition group, which spent significantly less time exploring social stimuli during the three‐chamber test (0.57 ± 0.04 social index) compared to saline‐injected controls (0.74 ± 0.03 social index, *p* < 0.01, Figure 1G). However, inhibiting oxytocinergic neurons during the late postnatal stage (P21–P27, after weaning) had minimal influence on autism‐associated behaviors, including repetitive behaviors and impaired social interaction (Figure 1E–G). These results suggest that oxytocinergic neurons are indispensable for developing social behavior in the early rather than late postnatal stage.

### 2.2 Early Postnatal Oxytocin Deficiency Existed in Autistic Mouse Brain

VPA exposure during pregnancy increases the risk of autism in humans,^[^ ^18^ ^]^ and VPA exposure in mice also induces neurodevelopmental abnormalities and deficits in social behaviors. In this study, we aimed to identify autistic pups as early as possible for early intervention. Consistent with previous reports,^[^ ^19^ ^]^ we found that the VPA‐exposed mice developed crooked tail features by P7, which became more pronounced by P14 (Figure S3A, Supporting Information). We chose these crooked‐tailed mice as the VPA‐exposed mice. We found no difference in body weight between the control group (Con) and the VPA‐exposed group (VPA) (Figure S3B, Supporting Information). Beginning at P30, we conducted behavioral tests associated with autism at 5‐day intervals. The VPA‐exposed mice showed significantly increased self‐grooming (Figure S3C, Supporting Information) and marble‐burying behaviors (Figure S3D, Supporting Information). Compared with the saline‐injected group, the VPA‐exposed group spent less time interacting with the social stimulus mice. Furthermore, their social indices were lower than those of the saline‐injected group (Figure S3E, Supporting Information). To evaluate anxiety levels, we performed an open field test and an elevated plus maze test on both the Con and VPA mice, but no significant difference was found between the two groups (Figure S3F–K, Supporting Information). These results confirmed the occurrence of autistic behaviors in the VPA‐exposed mice.

**Figure 2:**
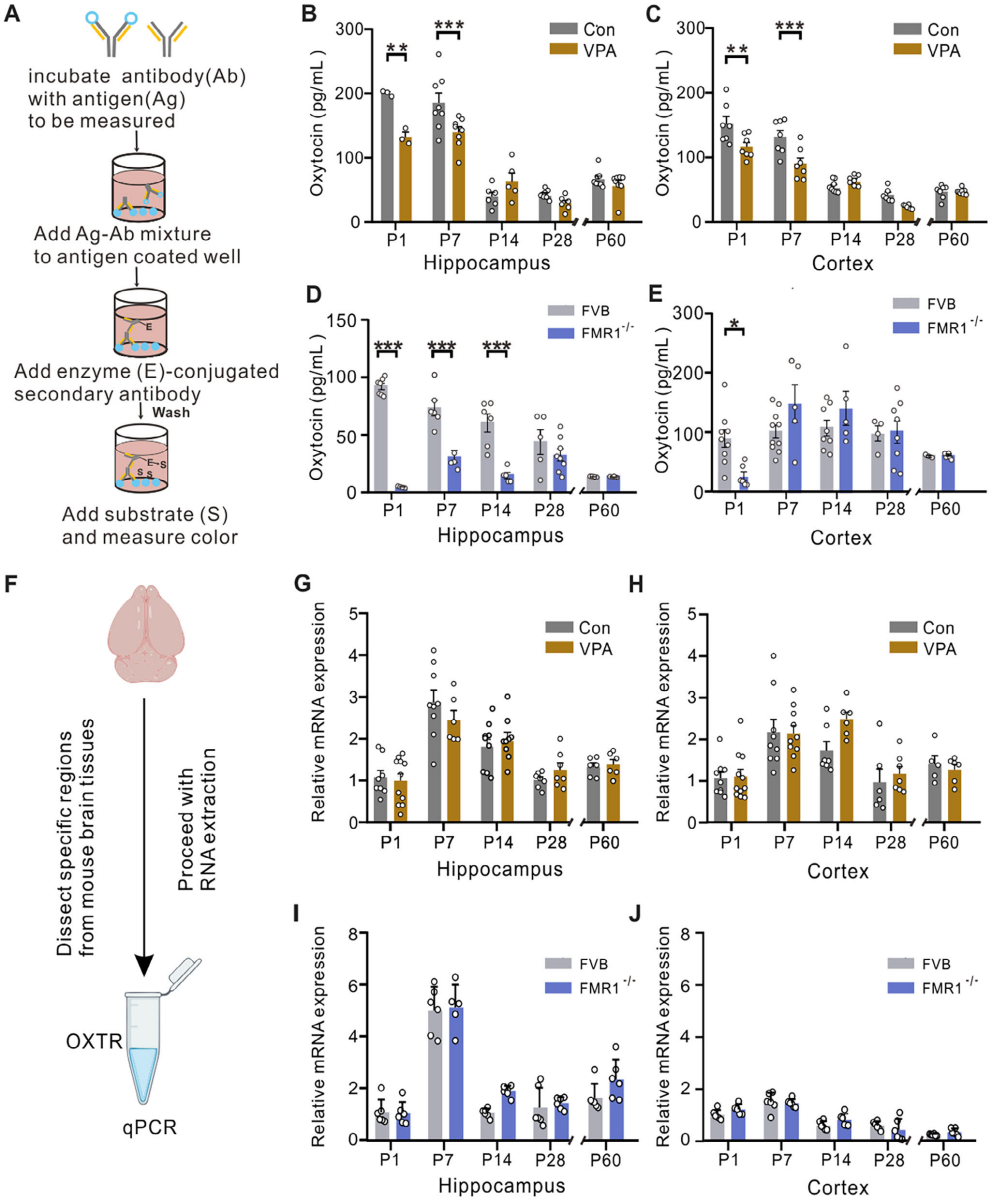
Oxytocin in the hippocampus and cerebral cortex of autistic mice was lower in the early postnatal stage. (A) Diagram of the competitive oxytocin ELISA. (B) Oxytocin content in the hippocampi of the control and VPA‐exposed pups [the hippocampal tissues of six P1 pups were combined into one sample, and three mixed P1 hippocampal samples were tested. **p < 0.01, P7, *n* = 8, ***p < 0.001, P14, control *n* = 6, VPA *n* = 5, *p* = 0.3508, P28, Con *n* = 8, VPA *n* = 6, *p* = 0.7823, P60, *n* = 8, *p* = 0.9535]. (C) Oxytocin content in the control and VPA‐exposed cortices (P1, *n* = 7; **p < 0.01; P7, *n* = 7; ***p < 0.001; P14, Con *n* = 8; VPA *n* = 7, *p* > 0.9999; P28, *n* = 6; *p* = 0.3335; P60, *n* = 8; *p* > 0.9999). (D) Oxytocin content in the hippocampi of the WT FVB and *Fmr1‐KO* pups (**p < 0.01, P7, FVB *n* = 6, *Fmr1‐KO n* = 5, ***p < 0.001, P14, FVB *n* = 6, *Fmr1‐KO n* = 5, ***p < 0.001, P28, FVB *n* = 5, *Fmr1‐KO n* = 8, *p* = 0.5395, P60, FVB *n* = 4, *Fmr1‐KO n* = 6, *p* > 0.9999). (E) Oxytocin content in the WT FVB and *Fmr1‐KO* cortices. (P1, FVB *n* = 9, *Fmr1‐KO n* = 6, *p < 0.05, P7, FVB *n* = 10, *Fmr1‐KO n* = 5, *p*  =  0.2149, P14, FVB *n* = 8, *Fmr1‐KO n* = 5, *p* =  0.6093, P28, FVB *n* = 4, *Fmr1‐KO n* = 8, *p* > 0.9999, P60, FVB *n* = 3, *Fmr1‐KO n* = 6, *p* > 0.9999). (F) Diagram of qRT‒PCR. (G) OXTR mRNA expression in the hippocampi of the control and VPA‐exposed pups (P1, Con *n* = 8; VPA *n* = 11, *p* = 0.9987; P7, Con *n* = 9; VPA *n* = 6, *p* = 0.4706; P14, Con *n* = 9; VPA *n* = 9, *p* = 0.9581; P28, *n* = 7, *p* = 0.9131; P60 *n* = 6, *p* > 0.9999). (I) OXTR mRNA expression in the control and VPA‐exposed pup cortices (P1, Con *n* = 9, VPA *n* = 11, *p* > 0.9999; P7, Con *n* = 9, VPA *n* = 10, *p* > 0.9999; P14, Con *n* = 7, VPA *n* = 6, *p* = 0.1171; P28, Con *n* = 6, VPA *n* = 7, *p* = 0.9798; P60, Con *n* = 5, VPA *n* = 6, *p* = 0.9979). (J) OXTR mRNA expression in the hippocampi of the WT FVB and *Fmr1‐KO* pups (P1, FVB *n* = 6; *Fmr1‐KO n* = 6, *p* > 0.9999; P7, FVB *n* = 6; *Fmr1‐KO n* = 5, *p* = 0.9992; P14, FVB *n* = 6; *Fmr1‐KO n* = 6, *p* = 0.0869; P28, FVB *n* = 6; *Fmr1‐KO n* = 6, *p* = 0.9919; P60, FVB *n* = 6; and *Fmr1‐KO n* = 6, *p* = 0.1990). (K) OXTR mRNA expression in the cortices of the WT FVB and *Fmr1‐KO* pups (P1, FVB *n* = 6; *Fmr1‐KO n* = 5, *p* = 0.5283; P7, FVB *n* = 6; *Fmr1‐KO n* = 6, *p* = 0.9986; P14, FVB *n* = 6; *Fmr1‐KO n* = 6, *p* = 0.4824; P28, FVB *n* = 6; *Fmr1‐KO n* = 6, *p* = 0.7058; P60, FVB *n* = 6; and *Fmr1‐KO n* = 6, *p* = 0.9625). The data are expressed as the means ± SEMs, and statistical significance is indicated as *p < 0.05 and **p < 0.01. Significance was determined using two‐way ANOVA.

We quantitatively measured the levels of oxytocin in the hippocampus and cerebral cortex at different developmental stages using ELISAs (**Figure** **2**A). Our results revealed a notable decline in hippocampal and cortical oxytocin levels during early postnatal development (Figure 2B,C). Physiologically, the oxytocin concentration sharply decreased by postnatal day 14, with the adult brain maintaining consistently low levels. Moreover, VPA exposure substantially reduced oxytocin levels in the hippocampus and cortex during the early postnatal stages (P1 and/or P7), but no significant difference was observed during the later developmental stages (P14, P28, and P60). To determine whether oxytocin deficiency also occurs in genetically mutated autistic mice, we quantified the oxytocin levels in *Fmr1*‐KO mice. Similar to the VPA‐exposed mice, the *Fmr1*‐KO mice also presented pronounced reductions in oxytocin levels in the early postnatal stages (Figure 2D,E).

Additionally, we examined oxytocin receptor expression at different developmental stages via RT‒qPCR (Figure 2F). The mRNA expression of the oxytocin receptor did not significantly differ between the Con and VPA‐exposed groups (Figure 2G,H) or between the WT FVB mice and the *Fmr1*‐KO FVB mice (Figure 2I,J). Collectively, our results suggest that oxytocinergic inputs may be severely affected in VPA‐exposed and *Fmr1‐*KO mouse brains during the early postnatal developmental stage.

### 2.3 GABA Reversal Potential was more Negative in VPA‐Exposed Brains During the Early Postnatal Stage

**Figure 3:**
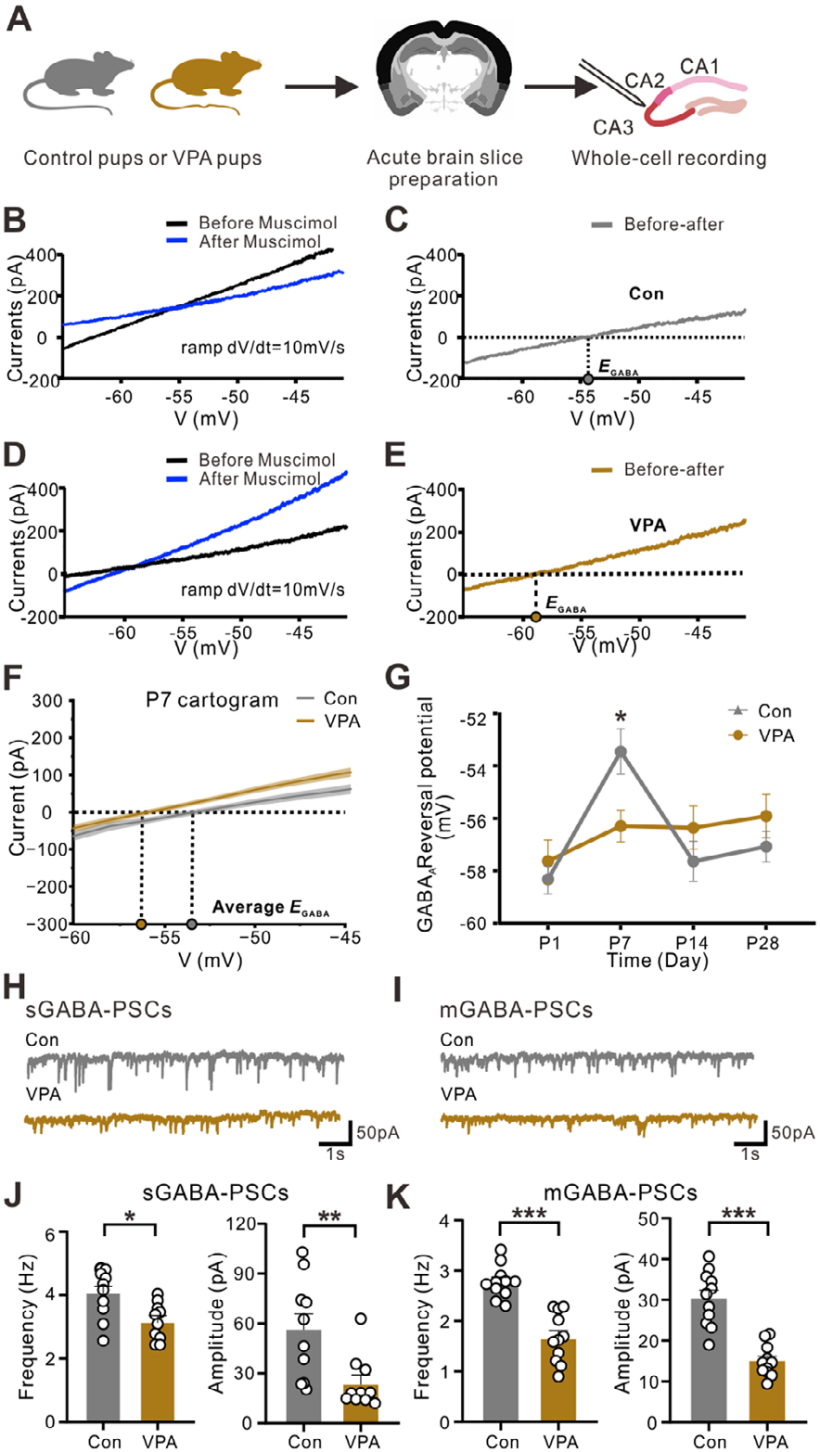
GABA_A_ reversal potential and GABA synaptic inputs were reduced in VPA‐exposed mice. (A) Diagram of whole‐cell patch clamp recording of the GABA_A_ reversal potential in CA3 pyramidal neurons in the hippocampus. (B) *I‒V* curves of GABA_A_ currents in control brain slices (before and after muscimol was applied). (C) Calculating the GABA_A_ reversal potential of the control brain (when the values of “Before” minus “After” equal zero). (D) *I‒V* curves of GABA_A_ currents in VPA brain slices. (E) Calculating the GABA_A_ reversal potential of the VPA brain. (F) Statistical results of the GABA_A_ reversal potential of the control and VPA‐treated mice at P7 (*n* = 4, *n* = 10). The mean value (line) and SEM (shadow area) of the *I‒V* curves are shown. (G) The GABA_A_ reversal potential at different time points. A significant difference was found in the VPA‐exposed mice at P7 (*n* = 4, *n* = 10) [ns. P1: *n* = 4, *n* = 9, *p* = 0.9330; P14: N = 4, *n* = 8, *p* = 0.6584; and P28 (N = 5, *n* = 9)]. The data are expressed as the means ± SEMs, and statistical significance is indicated as *p < 0.05 and **p < 0.01; two‐way ANOVA was used. (H) Representative sGABA‐PSC recordings in CA3 pyramidal neurons from the control and VPA‐treated mice. (I) Representative mGABA‐PSCs in CA3 pyramidal neurons from the control and VPA‐treated mice. (J) The frequency (left) and amplitude (right) of sGABA‐PSCs in CA3 pyramidal neurons at P7 were compared between the control mice (N = 5, *n* = 10) and the VPA‐treated mice (N = 4, *n* = 10). (K) The frequency (left) and amplitude (right) of mGABA‐PSCs in CA3 pyramidal neurons at P7 were compared between the control mice (N = 5, *n* = 11) and the VPA‐treated mice (N = 6, *n* = 11). The data were analyzed, with N denoting the number of mice and n indicating the number of recorded neurons. The data are expressed as the means ± SEMs, and statistical significance is indicated as *p < 0.05, **p < 0.01, and ***p < 0.001, unpaired *t*‐test. See also Figure S4 (Supporting Information).

Previous studies have shown that during childbirth, maternal oxytocin crosses the placenta, enters the fetal brain, and triggers the GABA polarity switch in the fetal brain.^[^ ^14^, ^15^ ^]^ However, it is unclear whether a postnatal deficiency in a pup's own oxytocin also influences the postnatal GABA reverse potential. We conducted whole‐cell patch clamp recordings of CA3 pyramidal neurons at various developmental stages (**Figure** **3**A). *I‒V* curves were obtained by recording the responses of the neurons in the control and VPA‐exposed mice to a slow ramp command (*dV/dt* = 10 mV s^−1^) before and after the application of the GABA_A_ receptor agonist muscimol (Figure 3B,D). The GABA_A_ receptor reversal potential was calculated by subtracting the two *I‒V* curves (Figure 3C,E). As shown in Figure 3F, the GABA_A_ receptor reversal potential of the VPA‐exposed pups was greater than that of the control pups, indicating a weakened depolarizing strength of GABA in the early postnatal stage. We observed a significant negative shift in the GABA_A_ receptor reversal potential in the VPA‐exposed mice at P7, but no differences were observed in the VPA‐exposed mice at P1, P14, or P28 (Figure 3G). We further recorded the spontaneous GABAergic postsynaptic currents (sGABA‐PSCs) in the Con and VPA groups at P7 (Figure 3H). We observed that both the frequency and amplitude of the sGABA‐PSCs were significantly decreased in the VPA‐exposed mice (Figure 3J). Similarly, we observed substantial decreases in the frequency and amplitude of miniature GABAergic postsynaptic currents (mGABA‐PSCs) (Figure 3I,K). Additionally, no significant changes were observed in the frequency or amplitude of spontaneous glutaminergic postsynaptic currents (sGlut‐PSCs) or miniature glutaminergic postsynaptic currents (mGlut‐PSCs) in the VPA‐exposed mice (Figure S4, Supporting Information). These data suggest that the GABA_A_ receptor reversal potential and GABAergic synaptic transmission, but not glutamatergic synaptic transmission, are significantly impaired in VPA‐induced autistic mice in the early rather than late postnatal stage.

### 2.4 Manipulating Oxytocinergic Neuronal Activity During the Early Postnatal Stage Significantly Altered the GABA_A_ Receptor Reversal Potential and Synaptic Transmission

**Figure 4:**
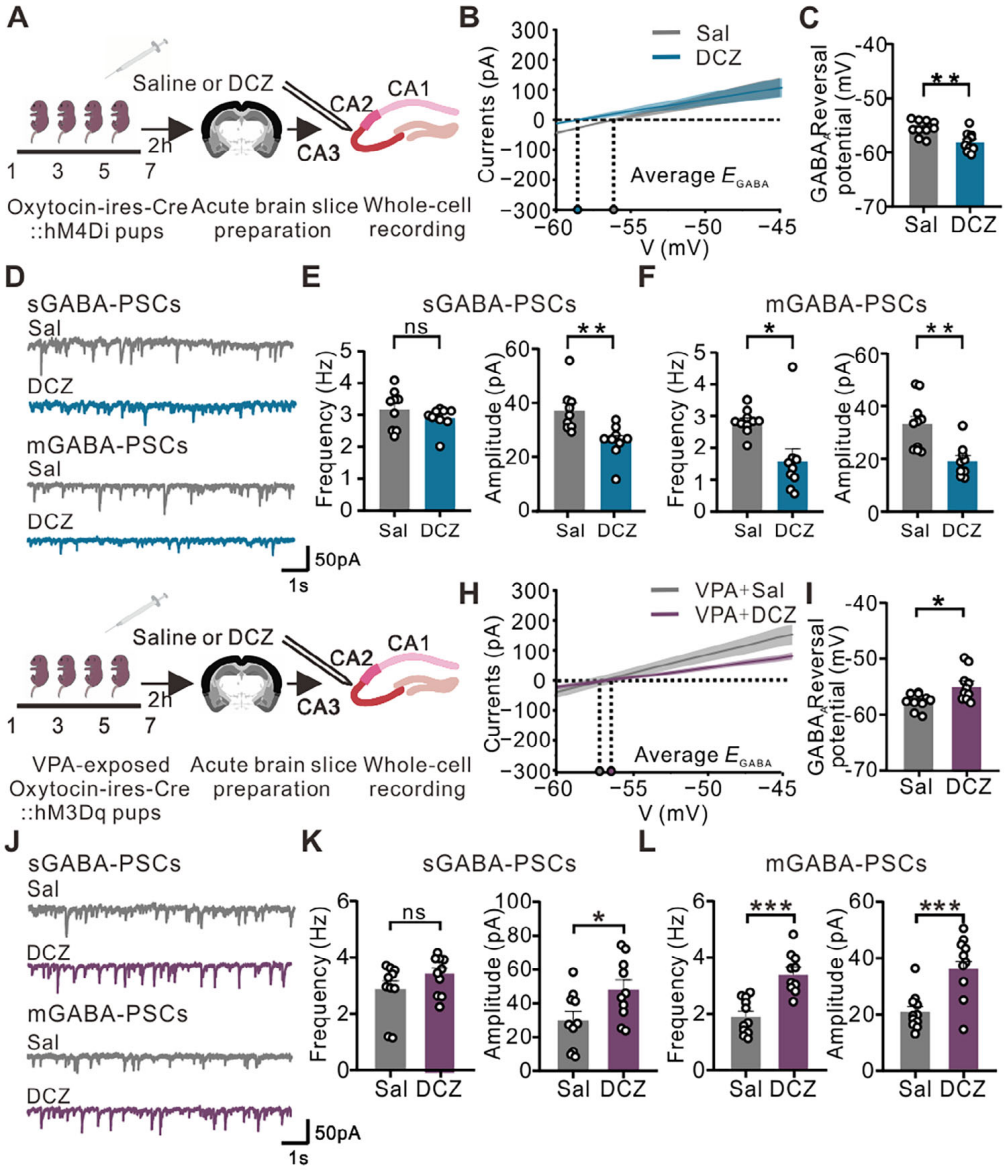
Chemogenetic inhibition or activation of oxytocinergic neurons modulates GABA_A_ receptor reversal potential and GABAergic synaptic inputs. (A) Schematic of the experimental design and paradigm of the experimental timeline. DCZ (or saline as a control) was injected into Oxt‐hM4Di mice at P1–P7, and 2 h later, acute brain slices were prepared at the last injection. CA3 pyramidal neurons were recorded with a whole‐cell patch clamp. (B) Statistical results of the GABA_A_ reversal potential of the saline‐ and DCZ‐treated mice at P7 (saline: N = 5, *n* = 10; DCZ: N = 4, *n* = 10). The mean value (line) and SEM (shadow area) of the *I‒V* curves are shown. (C) GABA_A_ reversal potential of CA3 pyramidal neurons after saline or DCZ delivery was recorded with a whole‐cell patch clamp (saline: N = 5, *n* = 10; DCZ: N = 4, *n* = 10; unpaired *t*‐test, **p < 0.01). (D) Representative sGABA‐PSC and mGABA‐PSC recordings of CA3 pyramidal neurons from the saline group and DCZ group (from top to bottom). (E) The frequency (left, *p* = 0.2694) and amplitude (right) of sGABA‐PSCs in CA3 pyramidal neurons at P7 were compared between the saline group (N = 5, *n* = 9) and the DCZ group (N = 4, *n* = 9). (F) The frequency (left) and amplitude (right) of mGABA‐PSCs in CA3 pyramidal neurons at P7 were compared between the saline group (N = 5, *n* = 10) and the DCZ group (N = 4, *n* = 9). (G) Schematic of the experimental design and paradigm of the experimental timeline. DCZ was injected (saline as a control) into OXT‐hM3Dq VPA‐exposed mice at P1–P7, and 2 h later, acute brain slices were prepared at the time of the last injection. CA3 pyramidal neurons were recorded with a whole‐cell patch clamp. (H) Statistical results of the GABA_A_ reversal potential of saline‐ and DCZ‐treated mice at P7 (saline: N = 4, *n* = 10; DCZ: N = 4, *n* = 10). The mean value (line) and SEM (shadow area) of the *I‒V* curves are shown. (I) CA3 pyramidal neuron GABA_A_ reversal potential after DCZ injection (saline as a control) was recorded with whole‐cell patch clamp (saline: N = 4, *n* = 10; DCZ: N = 4, *n* = 10; unpaired *t*‐test, *p < 0.05). (J) Representative sGABA‐PSC and mGABA‐PSC recordings of CA3 pyramidal neurons from saline‐treated mice and DCZ‐treated mice (from top to bottom). (K) The frequency (left, *p* = 0.1442) and amplitude (right) of sGABA‐PSCs in CA3 pyramidal neurons at P7 were compared between the saline group (N = 5, *n* = 10) and the DCZ group (N = 5, *n* = 10). (L) The frequency (left) and amplitude (right) of mGABA‐PSCs in CA3 pyramidal neurons at P7 were compared between the saline group (N = 5, *n* = 11) and the DCZ group (N = 6, *n* = 10). The data are expressed as the means ± SEMs, and statistical significance is indicated as *p < 0.05, **p < 0.01, and ***p < 0.001. Significance was determined via an unpaired *t*‐test. The data were analyzed, with N denoting the number of mice and n indicating the number of recorded neurons.

To explore the impact of modulating oxytocinergic neurons on GABA_A_ receptor reversal potential and postsynaptic currents, we conducted whole‐cell patch clamp recordings of CA3 pyramidal neurons at P7, 2 h after the final DCZ injection to the Oxt‐hM4Di pups (**Figure** **4**A). Inhibiting oxytocinergic neurons led to a significant negative shift in GABA_A_ receptor reversal potential induced by chemogenetic suppression of oxytocinergic neurons at P7 (Figure 4B,C), which is very similar to that in the VPA‐exposed mice. Furthermore, the amplitude of the sGABA‐PSCs was significantly reduced, and both the frequency and amplitude of the mGABA‐PSCs were significantly decreased (Figure 4D–F).

In addition to inhibiting oxytocinergic neurons, we also assessed the electrophysiological effect of chemogenetic activation of oxytocinergic neurons in VPA‐exposed mouse brains during the early postnatal stage (P1–P7) (Figure 4G). Chemogenetic activation of oxytocinergic neurons during early postnatal development restored the GABA_A_ receptor reversal potential in VPA‐exposed brains (Figure 4H,I). The activation of oxytocinergic neurons also increased the amplitude of the sGABA‐PSCs, as well as the frequency and amplitude of the mGABA‐PSCs (Figure 4J–L). These results highlight the potential of endogenous oxytocin to ameliorate the impaired electrophysiological properties of hippocampal CA3 pyramidal neurons at the early postnatal stage.

### 2.5 Chemogenetical Activation of Oxytocinergic Neurons or Oxytocin Supplement in Early Postnatal Development Ameliorated Autism‐Like Behaviors

**Figure 5:**
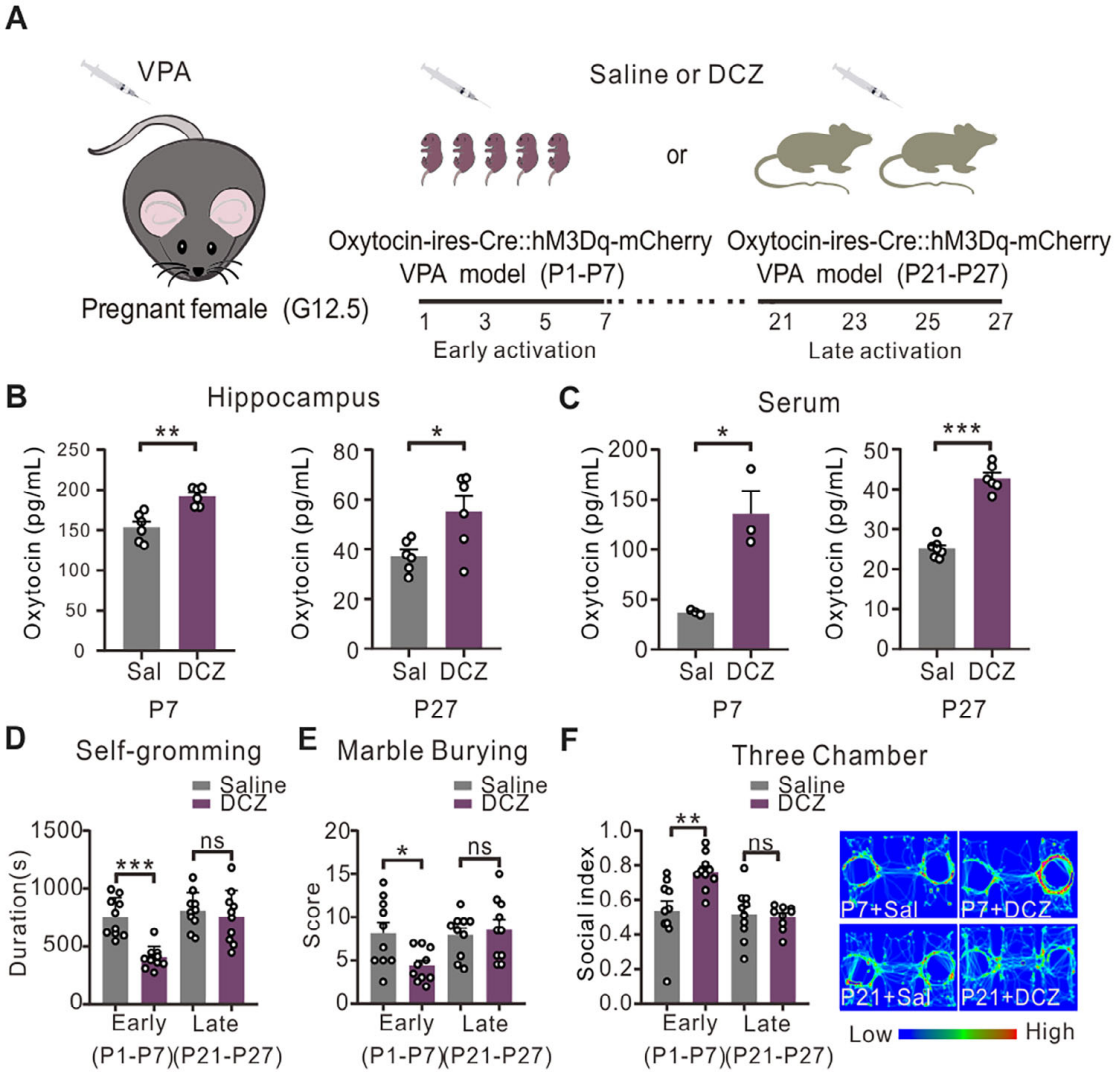
Activating oxytocinergic neurons from P1 to P7 ameliorated the autistic‐like behaviors of VPA‐exposed mice. (A) Paradigm of oxytocinergic neuron activation at the early postnatal stage (P1–P7) or late postnatal stage. DCZ was injected at different times to activate the OXT system (early treatment: P1–7; late treatment: P21–27) in the OXT‐hM3Dq VPA‐exposed group. (B) Hippocampal oxytocin contents after DCZ injection, P7 (*n* = 6, unpaired *t*‐test, **p < 0.01), and P27 (*n* = 6, unpaired *t*‐test, *p < 0.05). (C) Serum oxytocin contents after DCZ injection (P7, owing to the limited volume, the serum samples of six pups were combined into one sample, *n* = 3, unpaired *t*‐test with Welch's test, *p < 0.05; P27, saline *n* = 7, DCZ *n* = 6, unpaired *t*‐test, ***p < 0.001). (D) Time spent by self‐grooming VPA‐treated mice with early DCZ injection (saline *n* = 10, DCZ *n* = 10, unpaired *t*‐test, ***p < 0.001); time spent by the late DCZ injection group (saline *n* = 10, DCZ *n* = 10, unpaired *t‐*test, ns, *p* = 0.5620). (E) Marble‐burying test, early‐injection group (saline, *n* = 10; DCZ, *n* = 10; unpaired *t*‐test, *p < 0.05); late‐injection group (saline, *n* = 10; DCZ, *n* = 10; unpaired *t*‐test, ns, *p* = 0.6680). (F) Three‐chamber social preference test, early‐injection group (saline, *n* = 10; DCZ, *n* = 10; unpaired *t*‐test, **p < 0.01); late‐injection group (saline, *n* = 10; DCZ, *n* = 9; unpaired *t*‐test, ns, *p* = 0.7784). The data are expressed as the means ± SEMs, and statistical significance is indicated as *p < 0.05, **p < 0.01, and ***p < 0.001. See also Figure S5 (Supporting Information).

To investigate the therapeutic potential of endogenous oxytocin in VPA‐exposed mice, we opted for chemogenetic activation of oxytocinergic neurons during early postnatal development (P1–P7). After DCZ injection, the pups were returned to their home cages, allowing the dams to retrieve them and provide normal maternal care. We also established another group in which DCZ was administered at a later stage (P21–P27) (**Figure** **5**A). DCZ administration in Oxt‐hM3DGq mice effectively activated oxytocinergic neurons, as evidenced by increased oxytocin concentrations in both the hippocampal tissue and peripheral circulation, regardless of treatment timing (early versus late postnatal, Figure 5B,C). Notably, behavioral improvements in VPA‐exposed mice were strictly dependent on the timing of intervention, with only early postnatal activation producing a significant amelioration of autism‐like phenotypes (Figure 5D–F). Furthermore, we subcutaneously administered synthetic oxytocin to *Fmr1*‐KO pups either during the early postnatal stage or during the late postnatal stage and investigated their performance. Like chemogenetically activating oxytocinergic neurons, early but not late oxytocin supplementation effectively alleviated autistic behaviors in the *Fmr1*‐KO pups (Figure S5, Supporting Information). These results demonstrate a critical period for behavioral effects of oxytocin, with early postnatal intervention producing significantly greater amelioration of social behavior compared to administration at later developmental stages.

### 2.6 Oxytocin Upregulated the Expression of NKCC1 and Positively Shift GABA Reversal Potential in VPA‐Exposed Mice

**Figure 6:**
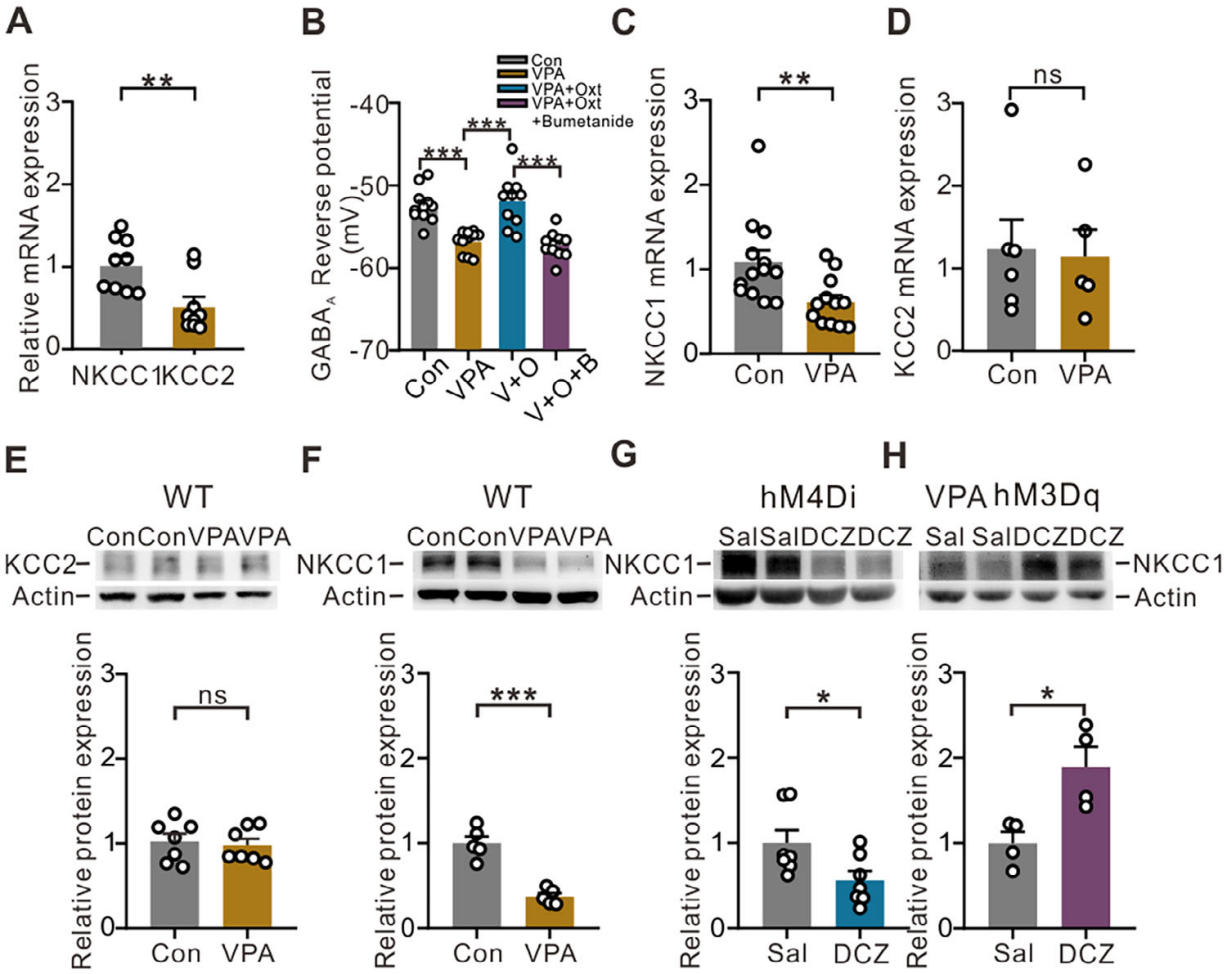
Oxytocin regulates GABA_A_ reversal potential by modulating the protein expression of NKCC1. (A) The mRNA expression levels of NKCC1 and KCC2 at P7 (the control group) were examined via RT‒qPCR (*n* = 9; unpaired *t*‐test; *p < 0.01). (B) The GABA_A_ reversal potential of P7 mice in the different treatment groups. The VPA + Oxt group received DCZ injections from P1 to P7. The GABA_A_ reversal potential of the VPA + Oxt + bumetanide group was recorded following bath application of bumetanide (10 µm, an NKCC1 antagonist). The Con (N = 7, *n* = 11), VPA (N = 7, *n* = 11), VPA + Oxt (N = 5, *n* = 10), and VPA + Oxt + bumetanide (N = 4, *n* = 10) groups were analyzed, with N mice and n recorded neurons. The data are expressed as the means ± SEMs, and the statistical significance is indicated as ***p < 0.001 (one‐way ANOVA + Tukey post hoc test). (C) The mRNA expression level of NKCC1 in different groups (Con and VPA) was examined via RT‒qPCR (NKCC1: Con N = 13, VPA N = 12, unpaired *t*‐test, **p < 0.01). (D) The mRNA expression level of KCC2 in different groups (Con and VPA) was examined via RT‒qPCR (Con, N = 6; VPA, N = 5; unpaired *t*‐test; ns, *p* = 0.8632). (E) The protein level of KCC2 in the control and VPA groups at P7 (Con *n* = 7, VPA *n* = 7, ns, *p* = 0.7136). (F) The protein level of NKCC1 in the Con or VPA group at P7 (*n* = 5, ***p < 0.001). (G) The protein level of NKCC1 in the saline‐injected or DCZ‐injected group of P7 OXT‐hM4Di mice (*n* = 7, *p < 0.05). (H) The protein level of NKCC1 in the saline‐injected or DCZ‐injected group of P7 OXT‐hM3Dq mice. (*n* = 4, *p < 0.05). The data are expressed as the means ± SEMs, and statistical significance is indicated as *p < 0.05, **p < 0.01, and ***p < 0.001. Significance was determined via an unpaired *t*‐test.

Tyzio et al. reported that oxytocin modulates the GABA_A_ reversal potential by regulating the chloride transporter.^[^ ^15^ ^]^ During the early postnatal stage, NKCC1 is the primary chloride transporter expressed in the central nervous system, while the expression of K^+^‐Cl^‐^ cotransporter 2 (KCC2) is significantly lower. Since both NKCC1 and KCC2 can regulate neuronal chloride concentrations but in opposite directions, we concurrently assessed the mRNA expression of NKCC1 and KCC2 in the hippocampus of P7 mice using qPCR. Our results revealed a significantly greater expression level of NKCC1 mRNA than of KCC2 mRNA (**Figure** **6**A). To investigate whether the effect of activating oxytocinergic neurons on the GABA_A_ receptor reversal potential is mediated by their regulation of NKCC1, we utilized a selective NKCC1 antagonist, bumetanide (10 µm), and observed that it effectively blocked the ameliorating effect of oxytocin (Figure 6B).

Next, we compared the mRNA expression of NKCC1 and KCC2 in the hippocampi of the control and VPA‐exposed mice. Our results revealed a significant decrease in NKCC1 mRNA expression and unchanged KCC2 mRNA expression in the VPA‐exposed mice (Figure 6C,D), indicating a downregulation of NKCC1 in the autistic‐like brain. To further confirm this result, we assessed the protein levels of KCC2 and NKCC1 at P7. Consistent with the mRNA expression results, we found no difference in KCC2 protein expression (Figure 6E), whereas there was a significant reduction in NKCC1 protein expression in the VPA‐exposed pups (Figure 6F). Furthermore, we investigated the impact of chemogenetic suppression of oxytocinergic neurons during early development (P1–P7) on the NKCC1 protein and detected a decrease in its protein level (Figure 6G). Conversely, selective activation of oxytocinergic neurons rescued the reduction in NKCC1 protein in the VPA‐exposed pups (Figure 6H). These results suggest that oxytocin plays a pivotal role in modulating the critical period of the GABA excitatory/inhibitory switch through the upregulation of NKCC1 expression in the early postnatal stage.

## 3 Discussion

Exploring the suitable time window of oxytocin treatment for ASD is crucial for guiding effective oxytocin treatments. In this study, we demonstrated that chemogenetically inhibiting oxytocinergic neurons during the early postnatal stage (P1–P7) significantly impaired social behaviors in mice. Furthermore, we observed a significant decrease in oxytocin levels, specifically during the early postnatal stage, in the brains of both drug‐exposed and genetic mouse models of autism. The ELISA results revealed a notable deficiency in oxytocin that was observed only during the early postnatal stage in those mouse brains, which significantly reduced NKCC1 expression and induced a negative shift in the GABA reversal potential, consequently impairing social behaviors in adolescence.

A meta‐analysis comprising a total of 1422 participants revealed that the endogenous oxytocin levels in children with ASD were significantly lower than those in neurotypical controls, whereas no difference was found in adolescent or adult populations. This preregistered meta‐analysis demonstrated that oxytocin deficiency mainly occurs in the early developmental stage, especially in children.^[^ ^20^ ^]^ Similarly, diminished oxytocin levels in the saliva of children with ASD in the morning but not in the afternoon were recently reported.^[^ ^21^ ^]^ Parker et al. reported that the benefits of oxytocin supplementation were more prominent in those children whose basal oxytocin levels in blood were low.^[^ ^22^ ^]^ A recent double‐blind, randomized, placebo‐controlled clinical trial reported that while oxytocin treatment did not affect the Social Responsiveness Scale‐2 (SRS‐2) score of all children, younger children who received oxytocin showed improvements in the SRS‐2 score.^[^ ^23^ ^]^ Furthermore, Le et al. reported that a 6‐week infrequent intranasal oxytocin treatment followed by a period of positive social interaction produces reliable symptomatic improvements in children (3–8 years) with ASD in terms of both social and repetitive behavior symptoms using the gold standard objective ADOS‐2 and SRS‐2.^[^ ^24^ ^]^ In this study, we checked oxytocin levels in mouse brains and reported that oxytocin deficiency in autistic mouse brains seems to occur only in the early postnatal stage. Therefore, both clinical and animal studies have suggested that oxytocin deficiency in the early postnatal stage might be a common pathophysiological mechanism in ASD, at least a partial part of ASD etiology.

In addition to analyzing brain tissues, we also examined circulating oxytocin levels. Our ELISA results revealed a progressive increase in circulating oxytocin (Figure S6, Supporting Information), while a significant developmental decline in oxytocin was observed in the hippocampus and cortex during early postnatal stages (P1 and/or P7, Figure 2B–E). Notably, the elevated oxytocin levels in the brain align with the peak expression of the oxytocin receptor (OXTR) during early development (Figure 2G–J), suggesting that high levels of oxytocin and its associated signaling pathways may be essential for proper neurodevelopmental processes.

The developmental reduction of oxytocin in the hippocampus and cortex is particularly intriguing, first, the mass or volume of hippocampus and cortex greatly expand during development, which would decrease the concentration of oxytocin. Second, as these regions do not synthesize oxytocin locally, their oxytocin content is likely derived from hypothalamic oxytocinergic neurons. This raises the possibility that the distinct changes in oxytocin levels in specific brain regions versus circulation may be attributed to the differential projection sources of oxytocinergic neurons. Li et al. (2024) provides valuable insights into the organization of oxytocinergic neurons in PVN. They reconstructed the axonal, dendritic, and somatic morphologies of individual oxytocin neurons and identified two distinct clusters of oxytocinergic neurons with unique morphological, transcriptional, and projection characteristics. Specifically, axons of cluster 1 neurons terminate exclusively in the median eminence (ME), while cluster 2 neurons project widely to multiple brain regions, but do not innervate the ME.^[^ ^25^ ^]^ Researchers have suggested that oxytocin plays a critical role in regulating various aspects of neurodevelopment and maturation after birth, which is referred to as the organizational effects of oxytocin on behaviors.^[^ ^26^ ^]^ Alterations in the oxytocin system during early development can have long‐term pathological consequences for rapid and dynamic brain development.^[^ ^27^ ^]^ For example, administering exogenous oxytocin to *Magel2*‐deficient (KO) mice from P0 to P6 was sufficient to restore deficits in social interactions in the adult stage.^[^ ^28^ ^]^

While our study focused on CA3 pyramidal neurons, notably, the autistic‐like behaviors likely arise from distributed neural network dysfunction. Our ELISA results (Figure 2) showed a significant reduction in oxytocin levels in the hippocampus and cortex, suggesting a reduced role of oxytocin in these regions. The literature indicates that the CA3 region plays an important role in memory formation and social interaction, as well as in the pathophysiology of autism spectrum disorder (ASD).^[^ ^29^ ^]^ Furthermore, altered GABA_A_ receptor reversal potential and GABAergic synaptic transmission are believed to contribute to the development of autistic‐like behaviors, particularly through mechanisms that affect neural network excitability and the balance between excitation and inhibition. Antoine et al. reported a common increase in the excitation‐inhibition conductance ratio across four genetic forms of ASD mouse models.^[^ ^30^ ^]^ In this study, we found abnormal GABA_A_ receptor reversal potential and impaired GABAergic synaptic transmission in the CA3 region during the early postnatal development stage. This abnormality is restricted to the early postnatal stage, making it challenging to specifically manipulate the GABA reversal potential, up‐regulate NKCC1, or selectively activate oxytocin afferent inputs to CA3 neurons to obtain direct evidence of causality within the first postnatal week. Our findings indicate that VPA‐exposed and *Fmr1*‐KO mouse models of ASD exhibit deficits in oxytocin levels and GABA reversal potential. Moreover, chemogenetic activation of oxytocinergic neurons or supplementation of oxytocin effectively restores the GABA reversal potential and improves autism‐like behaviors. These results suggest that impaired GABA reversal potential and GABAergic synaptic transmission in CA3 play a critical role in the development of autistic behaviors.

Similar brain development processes, including neurogenesis, neuronal apoptosis, myelination, synaptogenesis, and synaptic pruning, are highly comparable in rodents and humans.^[^ ^31^ ^]^ Notably, developmental time scales and brain maturation rates are known to vary across species. Compared with humans, mice are immature at birth and experience accelerated postnatal development, with the primary period of brain growth occurring in the first postnatal week.^[^ ^32^ ^]^ Given this timeline and our current findings, the timing of oxytocin use in ASD should be further studied in humans, whose brains are more mature at birth. Given that ASD diagnosis occurs around the age of 3, the potential benefits of early oxytocin supplementation before an ASD diagnosis are a subject that warrants careful consideration and further exploration; oxytocin could be considered for children identified as being at high risk of autism.

Tyzio et al. initially identified a maternal oxytocin‐mediated excitatory‐to‐inhibitory GABA polarity switch during parturition, which serves to protect the neonatal brain during delivery.^[^ ^14^ ^]^ A blunted GABA polarity switch during delivery has been linked to autism‐related pathologies.^[^ ^15^, ^33^ ^]^ Importantly, we detected a peak in the driving force of GABA rebound to a high level at P2 in the control rats after parturition. In contrast, the driving force of GABA was significantly negative in the brains of the VPA‐exposed rats. Similarly, we observed a significantly reduced GABA_A_ receptor reversal potential in the VPA‐exposed mouse brains at P7. This finding suggests that the postnatal decrease in the GABA_A_ receptor reversal potential in autistic brains after birth is noteworthy and important, given that this abnormality is concurrent with the time when the neonatal oxytocin system starts to play a critical role in social development, whereas oxytocin deficiency occurs only during the early postnatal stage.

The depolarizing and excitatory role of GABA during early postnatal neurodevelopment is attributed to the increased expression ratio of NKCC1/KCC2.^[^ ^34^ ^]^ During early postnatal development, NKCC1 is the predominant chloride cotransporter in the brain, exhibiting higher activity than KCC2. Specifically, we found that NKCC1 mRNA levels were markedly lower in the brains of the VPA‐exposed mice than in those of the control mice, whereas KCC2 expression was not significantly changed. Following chemogenetic activation of oxytocinergic neurons, NKCC1 protein levels are notably increased in the hippocampus. We hypothesize that oxytocin, released from activated oxytocinergic neurons, binds to its receptors and activates downstream signaling pathways, enhancing gene transcription and thereby increasing NKCC1 mRNA levels, which subsequently leads to elevated protein levels in the hippocampus. Supporting this, the literature indicates that in the outer medullary collecting duct, in vivo administration of oxytocin significantly increases NKCC1 mRNA levels.^[^ ^35^ ^]^ In addition to NKCC1, it has been documented that oxytocin modulates KCC2 membrane trafficking and stability in an Oxtr‐dependent manner, via the phosphorylation of KCC2.^[^ ^36^ ^]^ Further research is needed to elucidate the precise molecular mechanisms involved and to determine whether similar regulatory dynamics occur during the early postnatal developmental stage.

A randomized, double‐blind, placebo‐controlled clinical studies indicated that bumetanide, a specific antagonist of NKCC1, effectively alleviated symptoms of autism,^[^ ^37^ ^]^ highlighting NKCC1 as a promising therapeutic target. However, two subsequent large‐scale phase III clinical trials failed to show superior clinical efficacy of bumetanide compared to placebo.^[^ ^38^ ^]^ These inconsistent results of clinical trials underscore the complexity of bumetanide treatment in ASD patients. It is possible that the timing or status of the GABA switch varies among different ASD patients, and this factor should be considered and confirmed before initiating bumetanide therapy.

The NKCC1 protein is expressed in multiple cell types of the brain,^[^ ^39^ ^]^ and its activity or expression rapidly changes during the early postnatal stage. Our study indicated that NKCC1 might be regulated by oxytocin and determine the reversal potential of the GABA_A_ receptor in the early postnatal stage. Because of the limitations of anti‐NKCC1 and anti‐KCC2 antibodies, this study did not resolve the identities of cells whose NKCC1 expression was influenced by VPA exposure and whether oxytocin cell‐specifically increased the expression of NKCC1. Therefore, further studies employing techniques such as single‐cell sequencing or single‐cell proteomics may be needed to examine changes at a cell‐specific resolution.

Recent reports indicate that oxytocin nasal spray treatment in combination with concomitant psychosocial training results in greater improvements in social responsiveness.^[^ ^40^ ^]^ In fact, oxytocin is believed to increase the salience of social stimuli, thereby promoting neural processes related to social responses.^[^ ^41^ ^]^ In this study, we activated oxytocinergic neurons from P1 to P7, and the pups were sent back to their dam, which might provide a normal social interaction environment and allow the oxytocin system to shape social behavior development effectively. Therefore, combining early oxytocin treatment with cognitive and behavioral therapy interventions is recommended.^[^ ^42^ ^]^

## 4 Conclusion 

Cumulatively, our findings suggest that oxytocin's impact on brain development might be more pronounced within a specific early postnatal timeframe, coinciding with the emergence of the GABA excitatory/inhibitory switch—a process likely to occur postnatally developmental process and possibly occurring only once throughout the whole lifespan. This perspective emphasizes the unique temporal window in which oxytocin influences the GABA excitatory/inhibitory switch during the early developmental stages within an individual's lifetime.

## 5 Experimental Section

### Animals and Genotyping

The B6;129S‐*Oxt^1.1(cre)Dolsn^*/J (Oxt‐IRES‐Cre) mice, B6.Cg‐Gt(ROSA)26Sor^3.3(CAG‐EGFP, CHRM3*/mCherry/Htr2a)Pjen^/J (hM3Dq) mice, B6.129‐Gt(ROSA)26Sor^1(CAG‐CHRM4*,‐mCitrine)Ute^/J (hM4Di) mice and FVB.129P2‐Pde6b^+^ Tyr^c‐ch^ Fmr1 ^tm1Cgr1^/J were ordered from the Jackson Laboratory (ME, USA). C57BL/6JGpt‐Oxt^em5Cd6850^/Gpt mice (strain no. T027317) were obtained from GemPharmatech LLC (Nanjing, CN). New strains of Oxt‐IRES‐Cre::Cre‐dependent chemogenetic mice were generated by crossing Oxt‐IRES‐Cre mice with hM3Dq or hM4Di mice. All the experimental procedures were performed according to the Laboratory Animal Care Guidelines and were approved by the Animal Ethical and Welfare Committee of Nanjing University. Efforts were made to minimize the number of animals used and the pain or distress experienced by the animals.

All the mice were genotyped by PCR starting from DNA extracted from tail snips (≈2 mm) and using the primers as follows:

Oxytocin‐KO‐mut:
F: TCTGTCTAGAAATGGCCCTTCTGT; R: AAGACTTCCAGTCTCTGAGCTCCA
Oxytocin‐KO‐wt:
F: CTGCTGCAAGGACCATGAGTGAA; R: ACAACCTGGGATTGCTGCCTAC
hM4Di‐mut:
F: TGCTGCTTCATGTGGTCGG; R: CCATTGGCTACTGGCTCTGC
hM4Di‐wt:
F: TCAGATTCTTTTATAGGGGACACA; R: TAAAGGCCACTCAATGCTCACTAA
Oxytocin‐ires‐Cre:
1: ACACCGGCCTTATTCCAAG; 2: TTTGCAGCTCAGAACACTGAC;
3: AGCCTGCTGGACTGTTTTTG
Fmr1‐KO:
1: CACGAGACTAGTGAGACGTG; 2: TGTGATAGAATATGCAGCATGTGA;
3: CTTCTGGCACCTCCAGCTT
hM3Dq‐mut:
F: TGTATCCAGGAGGAGCTGATG; R: GGAGCAACATAGTTAAGAATACCAG
hM3Dq‐wt :
F: CAGGACAACGCCCACACA; R: AAGGGAGCTGCAGTGGAGTA

### Mouse Model

For exposure of the pups to VPA, a single dose of 600 mg kg^−1^ VPA (Sigma‒Aldrich, St. Louis, MO, USA) was carefully injected into the abdominal cavity of pregnant mice on embryonic day 12.5 (E12.5). The control group received sterile saline (0.1 mL for every 10 g of body weight). As previously reported, a new DREADD agonist, DCZ, was used to activate or inhibit DREADDs.^[^ ^43^ ^]^ Freshly prepared DCZ (MCE, NJ, USA) saline solution was administered intraperitoneally to the mice as a single injection at a 100 µg kg^−1^ dose (0.1 mL/10 g body weight), and saline solution was administered to the control group (the injection time and frequency were the same as those of the experimental group).

### Enzyme‐Linked Immunosorbent Assay (ELISA)

For measurement of plasma oxytocin levels, blood was collected from each mouse and centrifuged at 4000 rpm for 15 min at 4 °C. Owing to the limited blood volume from P1 to P14 pups, the blood samples were combined to obtain a sufficient plasma volume. Oxytocin levels were determined using an ELISA kit (Enzo Life Sciences, NY, USA, cat. number: ADI‐900‐153A) in accordance with the manufacturer's instructions. For measurement of oxytocin levels in the brain, control and VPA‐exposed mice at different developmental stages (P1, P7, P14, P28, and P60) were deeply anesthetized with isoflurane and sacrificed. The hippocampal and cerebral cortex tissues were promptly dissected, frozen in liquid nitrogen, and stored at −80 °C. The brain samples were weighed and then homogenized on ice with a Dounce tissue grinder using a neuronal protein extraction reagent (N‐PER, Thermo Fisher Scientific, MA, USA, cat. number: 87792) in the presence of a protease inhibitor (Protease Inhibitor Cocktail Tablets, Roche, Basel, Switzerland). The oxytocin levels in each sample were subsequently determined using OXT ELISA kits following the manufacturer's instructions. The total oxytocin content of each sample was expressed in pg mL^−1^.

### Behavior Tests

For all the behavioral tests, the mice were transported in their home cage to the testing room, where they habituated for 1 h before being placed in the testing arena. All testing was conducted between 09:00 and 12:00 h to avoid circadian cycle variability.

### Self‐Grooming

The self‐grooming behavior test was carried out as previously reported.^[^ ^44^ ^]^ The behaviors were videotaped and scored for the duration of self‐grooming behaviors during the subsequent 1 h period. The repeated stereotyped movements known as syntactic chains (elliptical stroke, unilateral stroke, bilateral stroke, and body licking) were considered typical self‐grooming behaviors. Usually, more flexible, nonchain self‐grooming behaviors were recorded, which were also considered grooming behaviors.^[^ ^40^, ^41^, ^42^, ^43^, ^44^, ^45^ ^]^ These self‐grooming behaviors were manually analyzed by different people who were blinded to the experimental groups.

### Marble‐Burying Test

The marble‐burying test was carried out as previously reported.^[^ ^46^ ^]^ Twenty black glass marbles (1.6 cm diameter) were placed on the bedding. The mice were placed in the cage and left undisturbed for 15 min. A Logitech c920 webcam was used to record the videos. After 15 min, the mice were removed from the cage, and the number of buried marbles was counted. The score was calculated as follows: more than 90% of the beads being buried received one point, 50–90% received 0.5 points, and less than 50% received zero points. Fresh litter was added after each test, and the glass beads were cleaned with 75% ethanol to prevent interference with the subsequent tests.

### Three‐Chamber Social Test

As reported previously,^[^ ^47^ ^]^ at the first stage, the mice were acclimated to the chambers with both sides empty for 10 min, and the mice were allowed to walk freely throughout all three chambers. In the second stage, a sociable mouse of the same sex and age was randomly placed in a wire cage in either chamber. For an additional 10 min, the test mouse was free to move in the device: a social chamber with the social stimulus mouse inside, a nonsocial empty side chamber, and a neutral middle chamber. During the test, video recordings were collected, and the amount of time spent in each chamber and the locomotion tracks were scored using the TopScan software. The social index was calculated as the percentage of time spent investigating the social target out of the total exploration time of both objects.

### Elevated Plus Maze (EPM) Test

The EPM test was carried out as previously reported.^[^ ^48^ ^]^ The labyrinth included four arms, each measuring 5 cm by 30 cm. There were two closed arms with walls 20 cm high and two open arms. The maze was set 50 cm above the ground. A tracking application (Clever TopScan) was used to monitor the movement of the mice for 5 min after they were positioned in the maze's center with their heads facing an open arm.

### Open Field Test

The open field test was carried out as previously reported.^[^ ^48^ ^]^ A video camera was used to record the spontaneous locomotor activities of the mice in the open field arena (50 × 50 × 50 cm). The Clever TopScan system (Clever Sys) was used to quantify the total distance traveled and the time spent by the mice in the center area for 5 min.

### Immunofluorescence Staining

Mouse brains tissue were collected after transcardial perfusion with phosphate‐buffered saline (PBS) and 4% paraformaldehyde (PFA) sequentially. For brain sections, mouse brains were fixed in 4% PFA for 24 h, followed by soaking in 15% and 30% sucrose solution for dehydration. For immunofluorescence staining, tissue sections were incubated in 5% bovine serum albumin (BSA) with 0.3% Triton‐X 100 in PBS for 1 h before incubation with primary antibodies (Oxytocin:1:500, Synaptic Systems, GER, cat. number: 408004, GFP:1:1000, Cell Signaling Technology, USA, cat. number: 2555S) at 4 °C overnight. Then, after three washes, the sections were incubated with appropriate fluorescent secondary antibodies (Oxytocin:1:1000, Anti‐Guinea pig IgG, Alexa Fluor 594 conjugated, Thermo Fisher Scientific, USA, cat. number: A‐11076, GFP:1:1000, Anti‐Rabbit IgG, Alexa Fluor 488 conjugated, Thermo Fisher Scientific, USA, cat. number: A‐11008) for 1 h at room temperature. Tissue was stained with DAPI (1:5000, Beyotime, CN) before image capture using a Leica TCS SP8‐MaiTai MP confocal microscope.

### RT‐qPCR

The cortex and hippocampus were acutely prepared and then freshly frozen in liquid nitrogen. Total RNA was extracted, quantified and reverse transcribed using SuperScript III RT SuperMix for qPCR (+gDNA wiper) (Vazyme, Nanjing, China, cat. number: R323‐01) according to the manufacturer's instructions. The mRNA expression of KCC2, NKCC1, OXTR, and GAPDH was quantified by real‐time PCR. The relative expression of OXTR, NKCC1, and KCC2 was normalized to that of GAPDH within the same sample. The final value is represented as a value relative to the control group.

KCC2:
F: GGGCAGAGAGTACGATGGC; R: CCCTGGGGTAGGTTGGTGTA
NKCC1:
F: GCAAGACTCCAACTCAGCCA; R: AAAGTAGCCATCGCTCTCCG
OXTR:
F: GTCTGGTCAAATACTTGCAGG; R: CGCGCAGCGAGAAAATGTG
GAPDH:
F: AACTTTGGCATTGTGGAAGGGCTCA; R: TTGGCAGCACCAGTGGATGCAGGG

### Electrophysiological Recordings

Coronal brain slices (300 µm in thickness) containing CA3 regions of the hippocampus were prepared with a vibroslicer (Leica, Wetzlar, Germany) in ice‐cold sucrose‐based slicing solution from mice of either sex. The sucrose‐based slicing solution contained the following (in mm): 212 sucrose, 3 KCl, 1.25 NaH_2_PO_4_, 26 NaHCO_3_, 7 MgCl_2_·6H_2_O, and 10 D‐glucose. Before recordings, the brain slices were incubated at 35 °C for 30 min in recovery solution containing the following (in mm): 118 NaCl, 2.6 NaHCO_3_, 11 glucose, 15 HEPES, 2.5 KCl, 1.25 NaH_2_PO_4_, 2 sodium pyruvate, 0.4 sodium ascorbate, 2 CaCl_2_, and 1 MgCl_2_. Then, the samples were maintained at room temperature for at least 1 h. The slices were transported to a submerged compartment and continuously superfused with artificial cerebrospinal fluid (ACSF) containing 125 NaCl, 25 NaHCO_3_, 2.5 KCl, 1.25 NaH_2_PO_4_, 11 glucose, 1.3 MgCl_2_, and 2.5 CaCl_2_ (in mm) at a rate of 2 mL min^−1^ while being kept at room temperature during recording sessions. All the solutions were constantly equilibrated with 95% O_2_ and 5% CO_2_.

For all the recordings, an Olympus BX51WI microscope (Olympus, Tokyo, Japan) was used to confirm the CA3 pyramidal neurons during the recording sessions. The data were amplified with an Axopatch‐700B amplifier (Axon Instruments, CA, USA), and the signals were low‐pass filtered at 1 kHz and digitized at 10 kHz with a Digidata‐1550 digitizer (Axon Instruments). The results were subsequently input into a computer for capture and analysis (pClamp 8.2, Axon Instruments). Neurons were excluded from the study if the series resistance was unstable or exceeded 20 MΩ. Borosilicate glass recording electrodes (3‐5 MΩ) were filled with either high Cl^−^ internal solution (composition in mm: 135 CsCl, 2 NaCl, 1 MgSO_4_, 0.2 CaCl_2_, 0.2 EGTA, 5 QX314, 4 Na_2_‐ATP, 0.4 Na‐GTP, adjusted to pH 7.2 with CsOH) or a Cs^+^‐methanesulfonate‐based internal solution (composition in mM: 120 cesium methanesulfonate, 20 CsCl, 10 HEPES, 0.2 EGTA, 10 sodium phosphocreatine, 5 QX314, 4 Na_2_‐ATP, 0.4 Na‐GTP, adjusted to pH 7.2 with CsOH) when the miniature or spontaneous postsynaptic currents were recorded. The K^+^‐methylsulfate‐based internal solution (composition in mm: 140 mm K‐methylsulfate, 7 mm KCl, 2 mm MgCl_2_, 10 mm HEPES, 0.1 mm EGTA, 4 mm Na_2_‐ATP, 0.4 mm GTP‐Tris, adjusted to pH 7.2 with KOH) was used when the driving force for Cl^−^ entry via the GABA_A_ receptors was examined.

The sGABA‐PSCs and mGABA‐PSCs were recorded at a holding potential of −70 mV using high Cl^−^ pipette solution in the presence of D‐APV (50 µm; Tocris Bioscience, Bristol, UK, cat. number: No. 0106/1) and NBQX (20 µm; Tocris Bioscience, Bristol, UK, cat. number: No. 0373/10), with or without TTX (0.3 µm; Alomone Labs, Israel, cat. number: 18660‐81‐6), respectively. Moreover, the sGlut‐PSCs and mGlut‐PSCs were recorded at a holding potential of −70 mV using a Cs^+^‐methanesulfonate‐based internal solution in the presence of SR95531 (20 µm; Tocris Bioscience, cat. number: No. 1262/10), with or without TTX (0.3 µm), respectively. For determination of the driving force for Cl^−^ to enter via GABA_A_ receptors, the equilibrium potential of Cl^−^ was evaluated in the slow‐ramp experiment (the voltage command ranged from −40 to −80 mV with dV/dt = 10 mV s^−1^) by comparing the *I‒V* curves before and after the application of muscimol (5 µm, Tocris Bioscience, cat. number: No. 0289/1), a highly selective agonist of GABA_A_ receptors. Bumetanide (10 µm, Tocris Bioscience, cat. number: No. 3108), a selective antagonist of NKCC1, was administered through a brief bath application (10 min) to examine the contribution of NKCC1 to the reversal potential of the GABA_A_ receptor.

### Oxytocin Administration

*Fmr1‐*KO pups (P1–P7) were removed from their mothers, placed on a heating pad, given a subcutaneous (s.c.) injection, and gently returned to the mother. The solutions injected were isotonic saline (10 µL) for control mice and 2 µg of OT (Phoenix Pharmaceuticals, Inc., cat #051‐01) diluted in isotonic saline (10 µL) for treated mice. For P21–P28, the solutions injected were isotonic saline (100 µL) for control mice and 20 µg of OT (Phoenix Pharmaceuticals, Inc., cat #051‐01) diluted in isotonic saline (100 µL) for treated mice.

### Western Blot

Protein samples were separated by SDS‒PAGE and then transferred onto Immobilon‐P membranes (Millipore, MA, USA). After the membranes were blocked in 5% nonfat powdered milk in Tris‐buffered saline with Tween‐20 (TBS‐T), they were probed with primary antibodies against the following proteins: NKCC1 (1:1000, rabbit polyclonal; Cell Signaling Technology, MA, USA, cat. number:14581), KCC2 (1:1000, rabbit polyclonal; Cell Signaling Technology, MA, USA, cat. number: 94725S), and β‐actin (1:1000, mouse polyclonal; Sigma, GER, cat. number: A1978‐200UL) at 4 °C overnight. The membranes were incubated with an anti‐rabbit/mouse IgG secondary antibody diluted at 1:2000 (Millipore) for 1 h, followed by enhanced chemiluminescence detection of the proteins with enhanced chemiluminescence (ECL) (Vazyme, Nanjing, CN). ImageJ software was used to assess the density of the immunoblots.

### Statistical Analysis

GraphPad Prism 8.0 was used for all statistical analyses. Sample sizes were chosen on the basis of previous studies conducted in the same field. All the data sets were first tested for normality before the statistical test was performed. Statistical significance was assessed by an unpaired Student's *t*‐test, an unpaired Student's *t*‐test with Welch's correction, one‐way ANOVA with Tukey's post hoc test, and two‐way ANOVA where appropriate. All values are presented as the means ± SEMs. The significance level was set at *p* < 0.05.

## Conflict of Interest

The authors declare no conflict of interest.

## Ethical Statement

All experiments were approved by the Animal Ethics and Welfare Committee of Nanjing University (IACUC‐2005012‐1). The experimental procedures were conducted in strict accordance with the National Institutes of Health Guide for the Care and Use of Laboratory Animals (revised in 2011).

## Supporting information



Supporting Information

## Data Availability

The data that support the findings of this study are available from the corresponding author upon reasonable request.
