# Supplementary material for: Oxytocin Improves Autistic Behaviors by Positively Shifting GABA Reversal Potential via NKCC1 in Early‐Postnatal‐Stage
Source: Adv Sci (Weinh). 2025 Apr 30;12(21):2415432. doi: 10.1002/advs.202415432 (PMC12140333; doi:10.1002/advs.202415432)
Supplement: Supplementary file 1 — Supporting Information [file ADVS-12-2415432-s001.docx]

Supporting Information

Oxytocin improves autistic behaviors by positively shifting GABA reversal potential via NKCC1 in early-postnatal-stage

*Zi-Hui Wang, Chang Xu, Yao-Yao Ma, Wei-Xuan Xue, Hao-Yuan Wang, Lin-Yao Fan, Chen-Yu Zhang, Liang Li*, Xiao-Yang Zhang*, Jing-Ning Zhu*, Qi-Peng Zhang**

*Correspondence to: qpzhang@nju.edu.cn (Qipeng Zhang)，jnzhu@nju.edu.cn (Jingning Zhu)， xiaoyangzhang@nju.edu.cn (Xiaoyang Zhang)， liangli@nju.edu.cn (Liang Li)


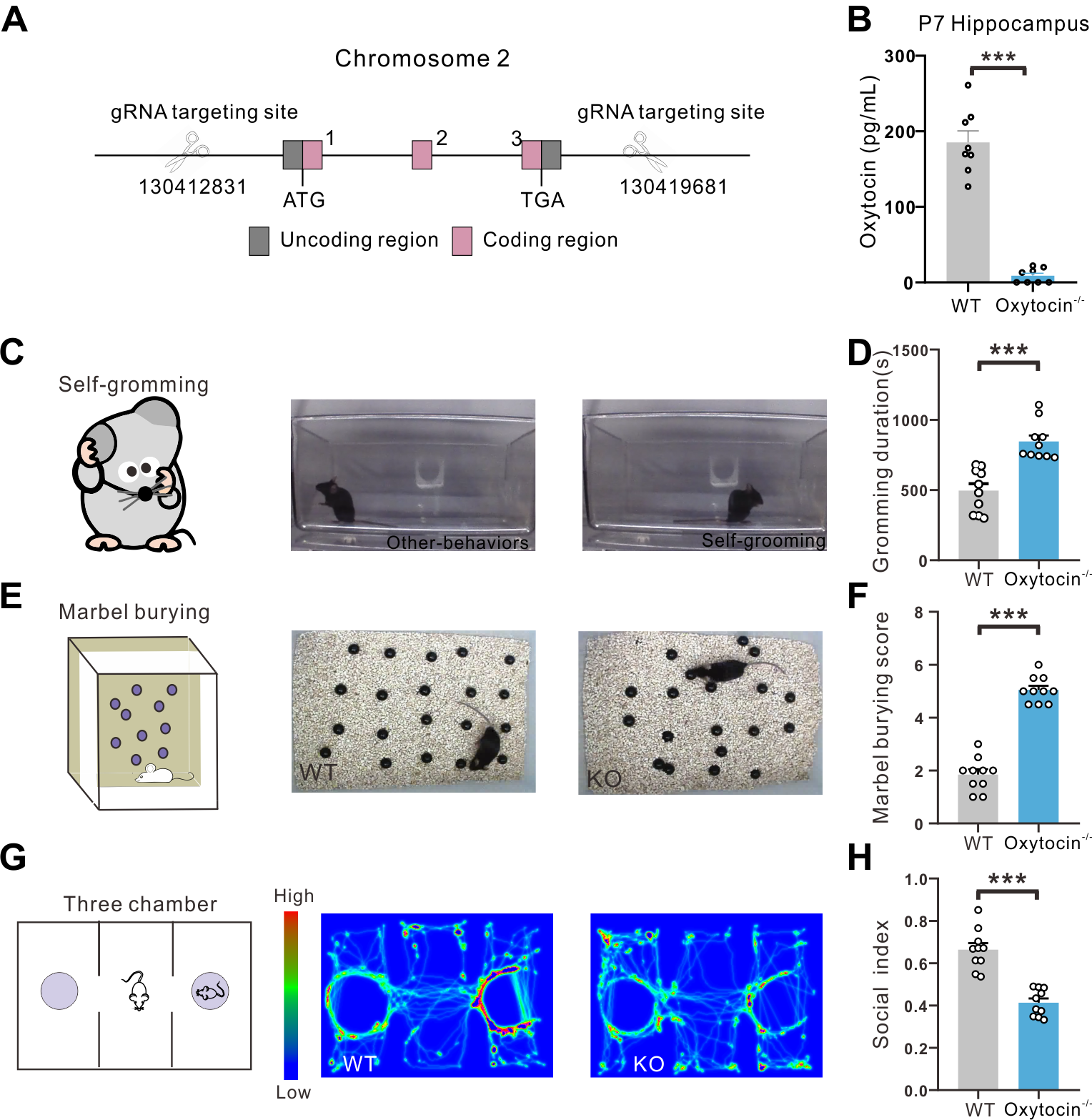


**Figure S1. Oxytocin knockout mice demonstrate characteristics of autism-like behaviors.** (A) Schematic representation of gene knockout. Oxytocin gene knockout was achieved by injecting oxytocin-targeted gRNA and Cas9 protein into zygotes. (B) Measurement of oxytocin levels in knockout mice. Compared to the control group, oxytocin levels in the hippocampal tissue of oxytocin gene knockout mice were significantly reduced at postnatal day 7 (n=8). (C) Schematic representation of self-grooming test (left), and comparison of grooming behavior with other behaviors (right). (D) Compared to the control group, oxytocin gene knockout mice showed a significant increase in self-grooming time (n=10). (E) Schematic representation of the marble burying test (left) and images of the end of the test for both types of mice (right). (F) Compared to the control group, oxytocin gene knockout mice displayed increased marble-burying behavior leading to a significant increase in marble-burying scores (n=10). (G) Schematic representation of the three-chamber social interaction test (left) and comparison of heat maps during the test (right). (H) Compared to the control group, oxytocin gene knockout mice exhibited a significant decrease in social preference (n=10).


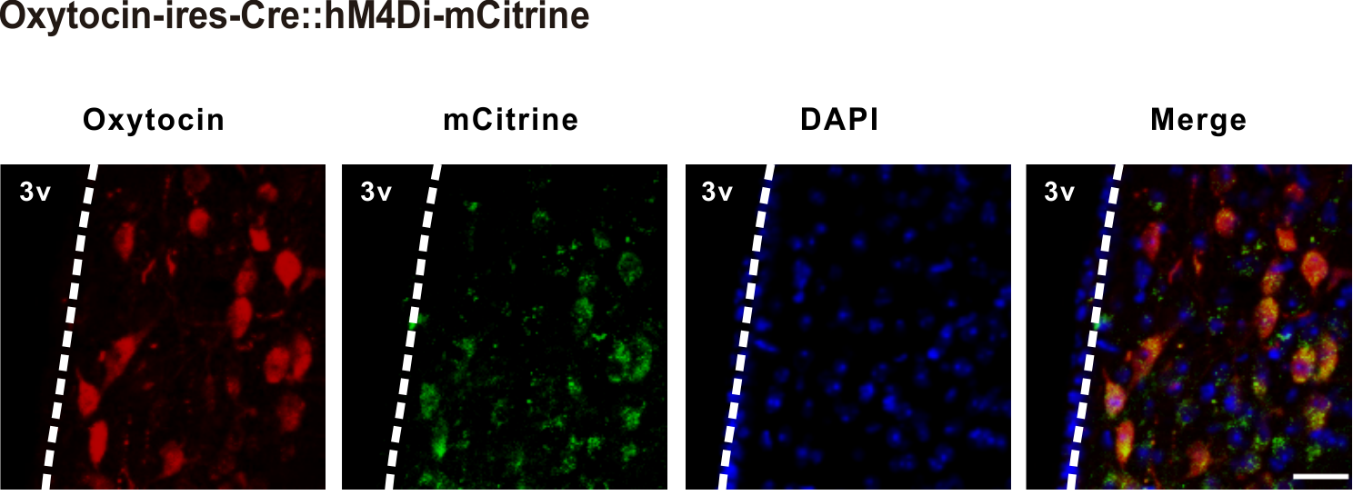


Figure S2. Immunohistochemical validation of mCitrine expression in Oxytocinergic neurons. Representative images of Oxytocin neuron (red) and mCitrine (green) fluorescence labeling in the PVN of Oxytocin-ires-Cre::hM4Di-mCitrine mice (Scale bar:25μm).


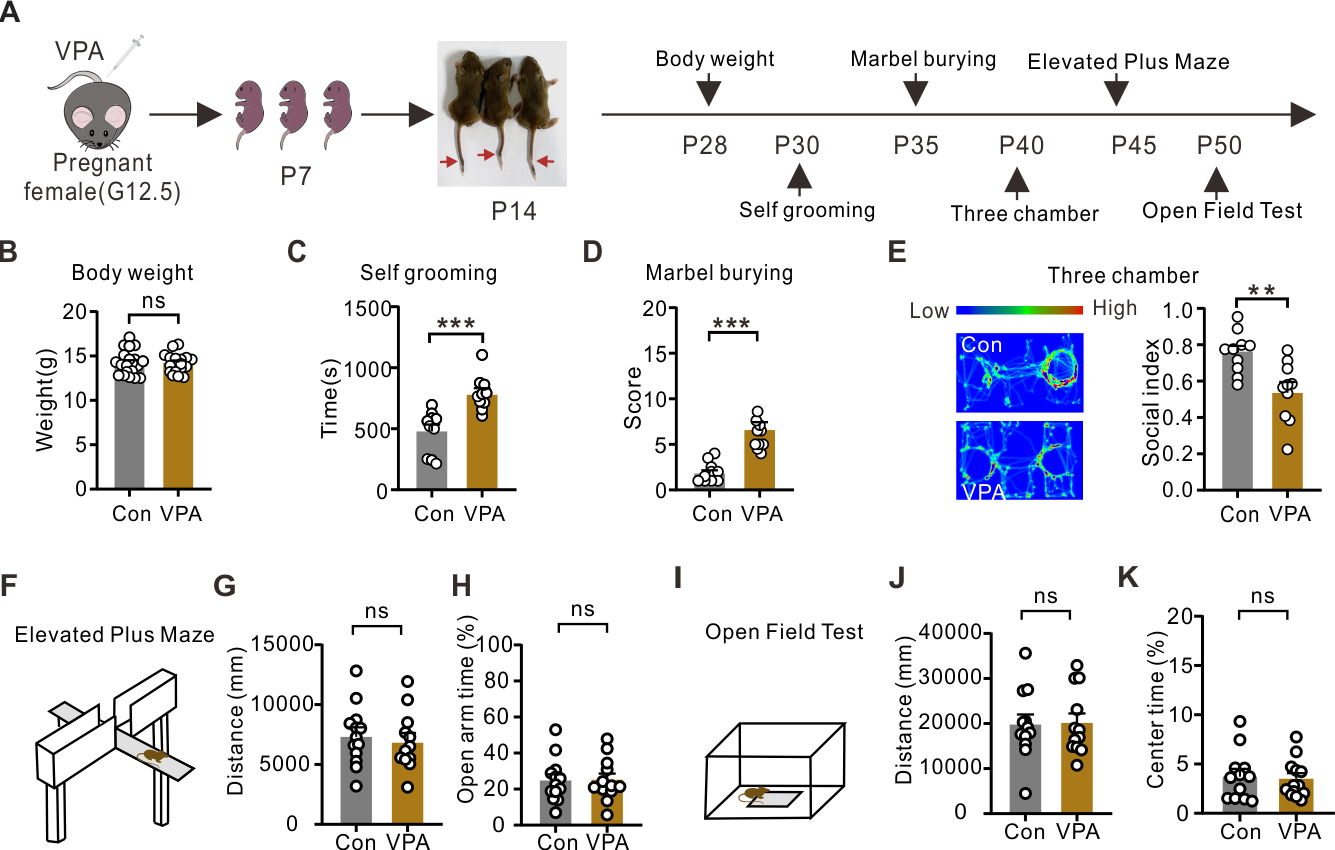


Figure S3. Behavioral Assessment of VPA-Exposed Mice. (A) Schematic of the experimental timeline. Pregnant female mice received the Saline or VPA injection at G12.5. The crooked tails in VPA-exposed mice were evident at P7 and became more obvious at P14. Three behavior tests were carried out at intervals of five days. (B) Body weights of both groups were measured at P28. (n=20, unpaired t-test, p=0.9599). (C) Self-grooming behavior test. (n=10, unpaired t-test, *** p < 0.001). (D) Marble-burying behavior test. (n=11, unpaired t-test, *** p < 0.001). (E) Three-chamber social preference test. Heatmaps of moving traits (left). (n=10, unpaired t-test, ** p < 0.01). (F) Schematic diagram depicting the Elevated Plus Maze tests. (G) Total movement distance during testing showed no difference between control and VPA-exposed mice (Con n=13, VPA n=12, unpaired t-test, ns, p=0.6284). (H) The proportion of open arm time during testing also showed no difference between control and model mice (Con n=13, VPA n=12, unpaired t-test, ns, p=0.9401). (I) Schematic diagram illustrating the open field tests. (J) Total movement distance during testing showed no difference between Con and model mice (n=12, unpaired t-test, ns, p=0.9063). (K) The proportion of center time during testing showed no difference between Con and VPA-exposed mice (n=12, unpaired t-test, ns, p=0.7621). Data are presented as mean ± SEM.


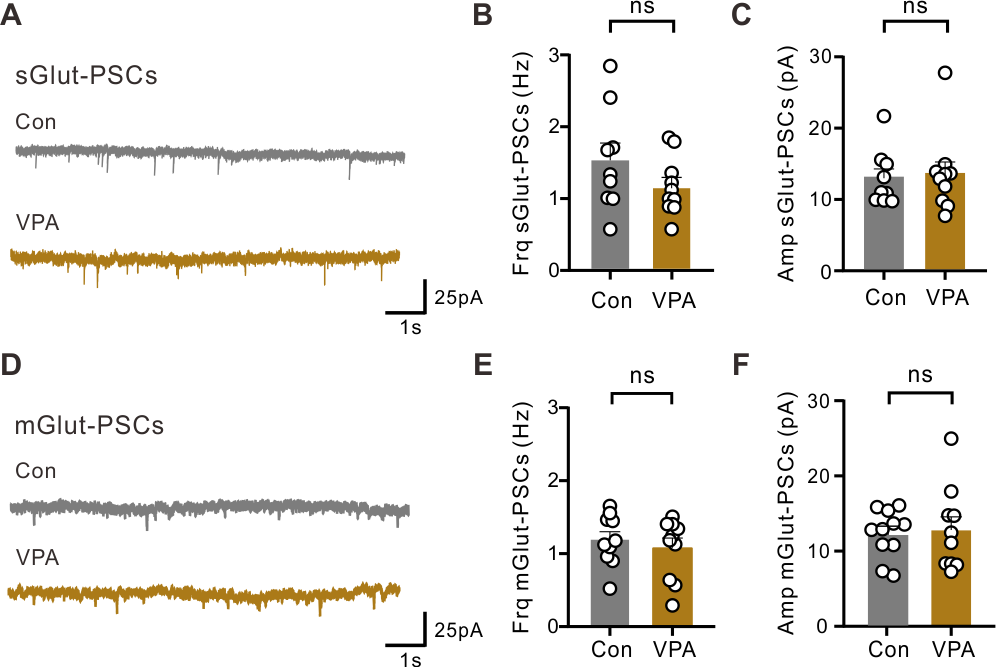


Figure S4. Lack of significant differences in Glutamatergic postsynaptic currents in VPA model mice. (A) Representative sGlut-PSCs in CA3 pyramidal neurons from the control group and VPA group. (B-C) The frequency (B) and amplitude (C) of sGlut-PSCs in CA3 pyramidal neurons at P7 were compared between the control mice (N=5, n=9) and VPA mice (N=6, n=10). No significant differences were observed in the amplitude (unpaired t-test, ns, p=0.8120) or frequency (unpaired t-test, ns, p=0.1761) of sGlut-PSCs between the VPA groups. (D) Representative mGlut-PSCs in CA3 pyramidal neurons from the control group and VPA group. (E-F) The frequency (E) and amplitude (F) of mGlut-PSCs in CA3 pyramidal neurons at P7 were compared between the control mice (N=5, n=10) and VPA mice (N=5, n=10). Similarly, no significant differences were found in the amplitude (unpaired t-test, ns, p=0.6818) or frequency (unpaired t-test, ns, p=0.4988) of sGlut-PSCs between the VPA groups. Data are presented as mean ± SEM, and statistical significance is denoted as *p < 0.05, **p < 0.01. Significance was assessed using an unpaired t-test. The data were analyzed with N denoting the number of mice and n indicating the number of recorded neurons.


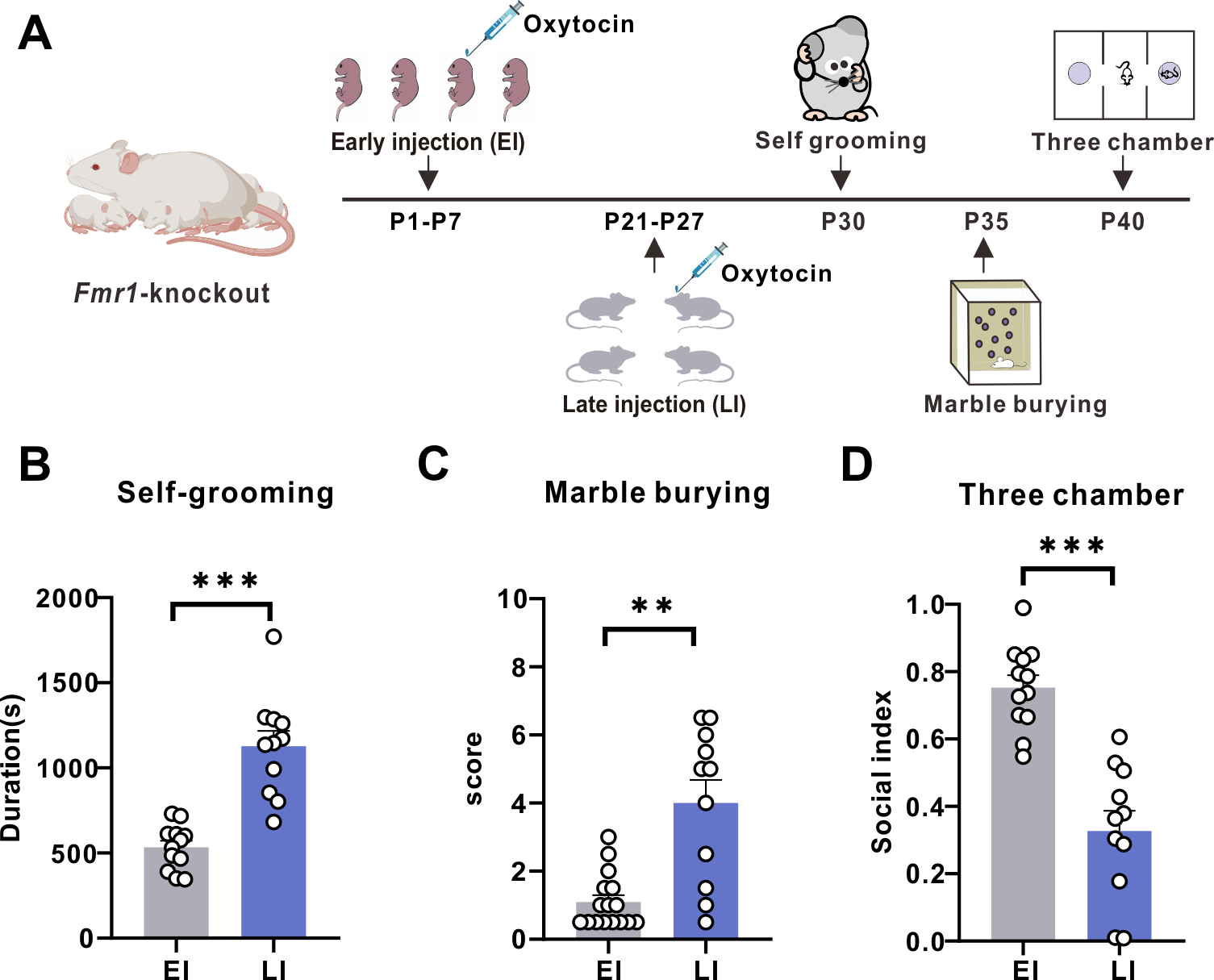


Figure S5. The administration of exogenous oxytocin to FMR1 knockout mice between postnatal days 1 and 7 (P1-P7) leads to an improvement in autistic-like behaviors. (A) Diagram of oxytocin treatment and behavioral testing protocol. (B) Time spent in self-grooming Fmr1-KO mice with early oxytocin injection (n=11) and the late oxytocin injection group (n=12, unpaired t-test, ***p<0.001). (C) Score in marble-burying test with early injection group and the late injection group (n=11, unpaired t-test, **p < 0.01). (D) Social index of two groups of mice in the Three-Chamber social preference test (n=10, unpaired t-test, ***p < 0.001). Data are expressed as the mean ± SEM and statistical significance is indicated as *p < 0.05, **p < 0.01, ***p < 0.001.


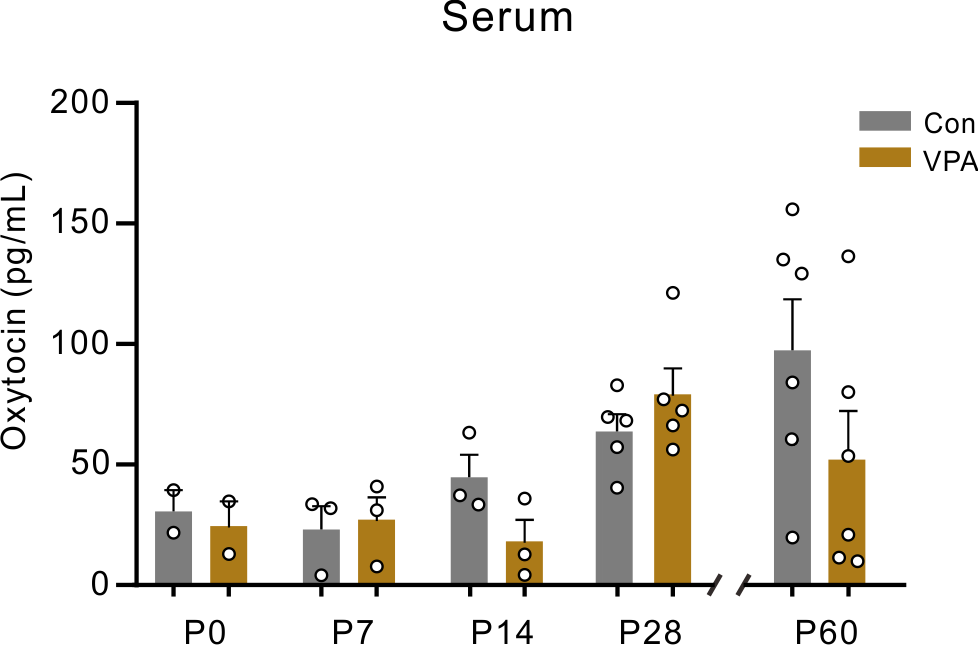


Figure S6. Detection of oxytocin levels in the serum of control and VPA-exposed mice. ELISA tests were performed at five time points: 0, 7, 14, 28, and 60 days postnatally in mice. Data are expressed as the mean ± SEM and there was no significant difference in serum oxytocin concentration between the control group and the VPA-exposed group.
